# Stem Cell Proliferation Is Kept in Check by the Chromatin Regulators Kismet/CHD7/CHD8 and Trr/MLL3/4

**DOI:** 10.1016/j.devcel.2019.04.033

**Published:** 2019-05-20

**Authors:** Louis Gervais, Marius van den Beek, Manon Josserand, Jérémy Sallé, Marine Stefanutti, Carolina N. Perdigoto, Patricia Skorski, Khallil Mazouni, Owen J. Marshall, Andrea H. Brand, François Schweisguth, Allison J. Bardin

**Affiliations:** 1 Institut Curie, PSL Research University, CNRS UMR 3215, INSERM U934, Stem Cells and Tissue Homeostasis Group, Paris, France; 2 Sorbonne Universités, UPMC Univ Paris 6, Paris, France; 3 The Gurdon Institute and Department of Physiology, Development and Neuroscience, University of Cambridge, Cambridge CB2 1QN, UK; 4 Menzies Institute for Medical Research, University of Tasmania, 17 Liverpool Street Hobart, Tasmania, 7000, Australia; 5 Institut Pasteur, Department of Developmental and Stem Cell Biology, Paris 75015, France; 6 CNRS, URA2578, Rue du Dr Roux, Paris 75015, France

**Keywords:** adult stem cells, tissue homeostasis, *Drosophila* midgut, chromatin regulators, Kismet/CHD7/CHD8, Trr/MLL3/4, EGFR, Epigenetic, Proliferation control, Cbl

## Abstract

Chromatin remodeling accompanies differentiation, however, its role in self-renewal is less well understood. We report that in *Drosophila*, the chromatin remodeler Kismet/CHD7/CHD8 limits intestinal stem cell (ISC) number and proliferation without affecting differentiation. Stem-cell-specific whole-genome profiling of Kismet revealed its enrichment at transcriptionally active regions bound by RNA polymerase II and Brahma, its recruitment to the transcription start site of activated genes and developmental enhancers and its depletion from regions bound by Polycomb, Histone H1, and heterochromatin Protein 1. We demonstrate that the Trithorax-related/MLL3/4 chromatin modifier regulates ISC proliferation, colocalizes extensively with Kismet throughout the ISC genome, and co-regulates genes in ISCs, including *Cbl*, a negative regulator of Epidermal Growth Factor Receptor (EGFR). Loss of *kismet* or *trr* leads to elevated levels of EGFR protein and signaling, thereby promoting ISC self-renewal. We propose that Kismet with Trr establishes a chromatin state that limits EGFR proliferative signaling, preventing tumor-like stem cell overgrowths.

## Highlights

• Chromatin modifiers Kismet and Trr limit intestinal stem cell proliferation
• Kismet and Trr colocalize at transcriptionally active regions and co-regulate genes
• EGFR negative regulator *Cbl* is a target gene of Kismet and Trr
• Kismet and Trr limit EGFR signaling in ISCs, preventing tumor-like ISC accumulation

## Graphical Abstract

**Figure:**
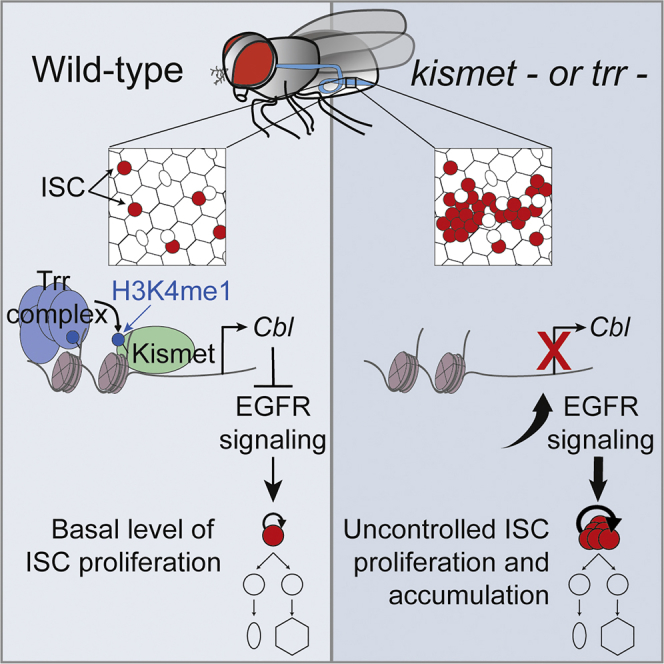

## Teaser

Stem cell proliferation control is essential to maintaining tissue homeostasis. Gervais et al. compare *Drosophila* intestinal stem cell (ISC) whole-genome profiling of chromatin modifier Kismet/CHD7/CHD8 binding to DamID reporters of different chromatin states and demonstrate that Kismet and Trr/MLL3/4 are regulators of ISC self-renewal by limiting EGFR signaling.

## Introduction

Regulation of stem cell proliferation rates is critical in adult tissues, which need to maintain basal renewal and undergo damage-induced regenerative responses. Consequently, the dysregulation of stem cell proliferation can have pathological effects. Ample evidence now supports a functional link between the deregulated proliferation of stem cells and cancer initiation, as well as metastatic progression (de Sousa e Melo et al., 2017, Flavahan et al., 2017). Interestingly, the loss of epigenetic control is a major contributor to stem cell misregulation including proliferation deregulation during aging (Brunet and Rando, 2017, Challen et al., 2014, Ko et al., 2011). Therefore, in addition to roles of epigenetic regulation during differentiation of stem-cell-derived lineages, chromatin modulation also has important, though not yet well understood, roles in the control of stem cell proliferation.

**Figure 1:**
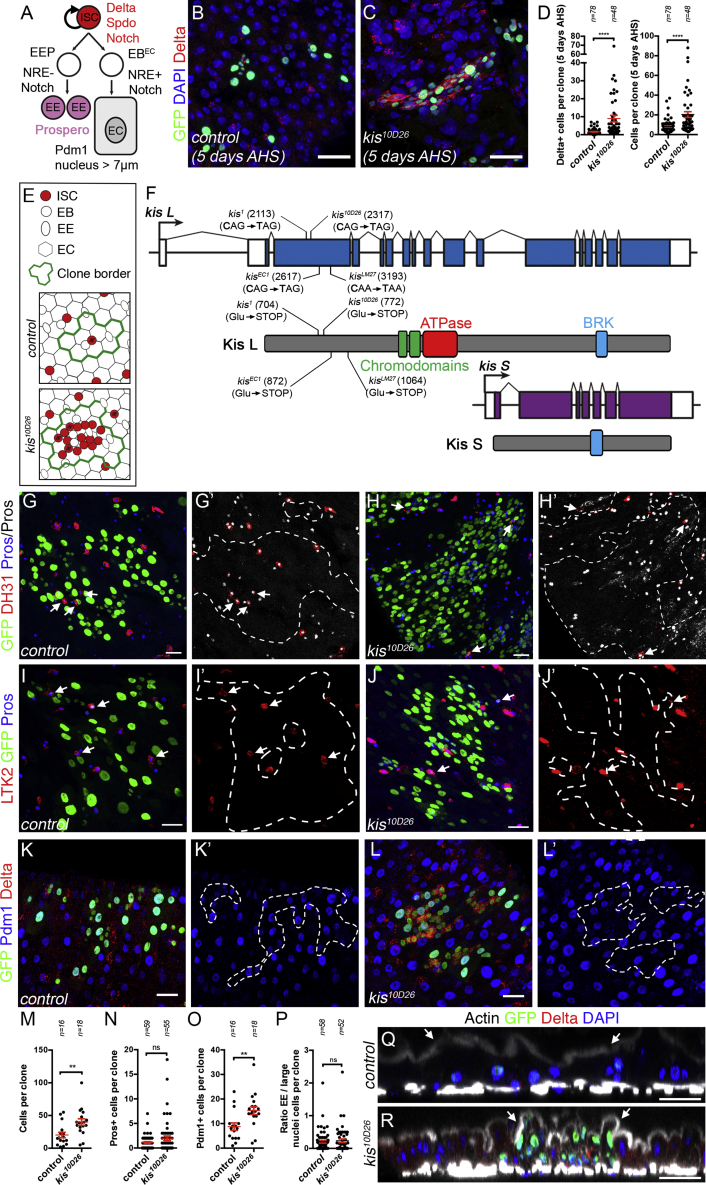
Loss of *kismet* Provokes ISC Accumulation without Affecting Terminal Differentiation (A) The ISCs divide to self-renew and to produce a precursor cell, the EB, that subsequently terminally differentiates into an EC or is thought to divide once as an EEP to produce two EE cells. (B and C) Wild-type (B) and *kis*^*10D26*^ mutant (C) MARCM clones, 5 days after heat shock (AHS). (D) Quantification of (B) and (C). (E) Scheme of wild-type and *kismet* mutant clones. (F) Scheme of *kismet* gene and Kismet protein (Long and short isoforms: Kis L and Kis S): chromodomains (green), ATPase domain (red), BRK domain (blue). All *kis* alleles resulted in nonsense mutations: nucleotide changes and corresponding putative resulting truncated proteins are shown. (G–L) Wild-type and *kis*^*10D26*^ MARCM clones at 9 days AHS. Arrows in (G)–(H′) and (I)–(J′) show EE cells marked by DH31 or LTK2, respectively. (M–P) Quantification of the total cells per clone (M), number of EE cells per clone (Prospero+) (N), number of ECs (Pdm1+ cells per clone) (O), and the ratio of EE (Prospero+ cells / EC (polyploid nucleus >7 μm) per clone (P). (Q and R) Vertical sections through the midgut epithelium of control (Q) and *kis*^*10D26*^ mutant (R) MARCM clones, 9 days AHS. Arrows show apical membrane. In (D) and (M)–(P), A two-tailed Mann-Whitney statistical test was used; mean values in red; error bars, SEM; ns = non-significant, **p < 0.01, ****p < 0.0001. Scale bars, 20 μm.

A useful model to investigate adult stem cell regulation is the *Drosophila* midgut, which is maintained by around 1,000 multipotent intestinal stem cells (ISCs). Most ISC divisions lead to asymmetric daughter cell fates, resulting in a self-renewed ISC and a sister enteroblast (EB) cell (Figure 1A). A majority of EBs receive high levels of Notch signaling and differentiate into enterocyte cells (ECs). Rare stem cell divisions produce an enteroendocrine precursor cell (EEP) with low or no Notch signaling, which is thought to divide once to make two enteroendocrine cells (EEs) (Chen et al., 2018, Ohlstein and Spradling, 2007, Sallé et al., 2017). In response to epithelial damage, several signaling pathways become activated and coordinate ISC proliferation and differentiation (see for review, Jiang et al., 2016). Of primary importance are signals that the ISCs receive to activate the Jak/Stat and Epidermal Growth Factor Receptor (EGFR) pathways (Biteau and Jasper, 2011, Buchon et al., 2010, Buchon et al., 2009, Jiang et al., 2011, Jiang et al., 2009, Wang et al., 2014, Xu et al., 2011). Moreover, other pathways such as Insulin, Hippo, Jun Kinase, BMP, Wnt, and Hedgehog also control ISC proliferation (Biteau et al., 2008, Cordero et al., 2012, Li et al., 2013, Li et al., 2014, Lin et al., 2008, O'Brien et al., 2011, Ren et al., 2010, Shaw et al., 2010, Staley and Irvine, 2010, Tian and Jiang, 2014, Tian et al., 2015, Tian et al., 2017). Evidence suggests that there are also mechanisms to limit ISC responsiveness, tuning down cell division when sufficient renewal has occurred (Guo et al., 2013, Hochmuth et al., 2011), though this process is not well understood.

Here, we report on the identification of a regulator that is essential to limit ISC proliferation: *kismet*, similar to the chromodomain-containing chromatin remodeling factors CHD7 and CHD8. Mammalian CHD7 is associated with transcriptionally active genes at enhancers, super-enhancers, and promoters where it can both activate and repress transcription (Hnisz et al., 2013, Schnetz et al., 2009, Schnetz et al., 2010). *kismet* was originally identified as a “trithorax group” gene because of its ability to dominantly suppress *Polycomb* mutant phenotypes (Kennison and Tamkun, 1988). Studies using the polytene chromosomes of the salivary gland have shown that Kismet is associated with transcriptionally active chromatin, where it recruits the histone methyl transferases Trithorax and Ash1. Ash1 recruitment, in turn, is thought to promote H3K36me2, leading to the inhibition of Histone H3K27me3 (Dorighi and Tamkun, 2013). In addition, as *kismet* mutants show a reduction in elongating RNA polymerase II (RNA Pol II), Kismet was proposed to promote transcription elongation (Srinivasan et al., 2008). Whether Kismet acts in other tissues via similar molecular effectors is not currently known.

CHD7/CHD8 and Kismet have essential functions during development and in adult tissues. In humans, inactivation of CHD7 causes a spectrum of congenital defects called CHARGE syndrome, (Coloboma, Heart defects, Atsresia choanae, Retarded growth and development, Genital abnormalities and Ear anomalies) (Bajpai et al., 2010, Vissers et al., 2004). Moreover, *CHD7* is essential for neural development and adult neural stem cell maintenance and is deregulated in cancers (Feng et al., 2013, Feng et al., 2017, Jones et al., 2015, Pleasance et al., 2010, Robinson et al., 2012). *CHD8* mutations are associated with neurodevelopmental defects in autism spectrum disorders (Neale et al., 2012, O’Roak et al., 2012, Talkowski et al., 2012). In *Drosophila*, *kismet* mutants have defects in developmental patterning with homeotic transformations as well as alteration of circadian rhythm and memory (Daubresse et al., 1999, Dubruille et al., 2009, Melicharek et al., 2010, Terriente-Félix et al., 2010, Terriente-Félix et al., 2011). However, to date, functions of *kismet* in stem cells have not been described.

Here, we demonstrate that Kismet is an important regulator of ISCs, essential to limit basal levels of ISC proliferation. DNA adenine methyltransferase identification sequencing (DamID-seq) of Kismet compared with DamID reporters of different chromatin states, including activated (Brahma and RNA Pol II) and repressed states (Polycomb, Histone H1, Heterochromatin Protein 1 [HP1]), demonstrated that Kismet preferentially localized to transcriptionally active regions of the genome and to developmental enhancers. In addition, our data suggest that Kismet acts in ISCs with the H3K4 monomethyltransferase, Trithorax-related complex (Trr; mammalian MLL3/4). We find that Trr and Kismet co-localize in the genome and co-regulate the transcription of many genes, including *Cbl*, a negative regulator of EGFR signaling. Our data therefore demonstrate that the chromatin regulators Kismet/CHD7/CHD8 and Trr complex/MLL3/4 function together to limit basal levels of ISC proliferation and identify *Cbl* as a key downstream target gene allowing control of EGFR signaling.

## Results

### Identification of *kismet* as an Essential Gene Controlling Stem Cell Homeostasis

We screened for EMS-induced mutations affecting ISC activity and intestinal homeostasis (C.P., F.S., and A.B., unpublished data) and found one line (10D26) that showed an increase in the size of mutant clones generated in the midgut of adult flies (Figures 1B and 1C) and had a higher proportion of cells expressing Delta, an ISC marker (8.9 Delta+ cells; 44% of clone) when compared to the control (1.6 Delta+ cells, 20% of clone; Figure 1D). In addition, these cells expressed the ISC marker Sanpodo (Perdigoto et al., 2011) (Figures S1A and S1B). Therefore, 10D26 mutant clones induce aberrant accumulation of stem cells (Figure 1E).

Deficiency mapping followed by failure to complement three known alleles of the *kismet* gene (*kismet*^*1*^, *kismet*^*EC1*^, and *kismet*^*LM27*^) indicated that 10D26 harbored a lethal mutation in *kismet*. *kismet* encodes a conserved chromatin remodeling factor, similar to *CHD7* and *CHD8* in mammals. *kismet* encodes a long (Kismet-L) and short (Kismet-S) isoform, with only Kismet-L containing two chromodomains and an SNF2-like ATPase/helicase domain, required for nucleosome remodeling activity (Bouazoune and Kingston, 2012) (Figure 1F). All four alleles correspond to nonsense mutations early in *kismet-L* coding sequence (Figure 1F). Clones for *kismet*^*EC1*^, *kismet*^*LM27*^, and *kismet*^*1*^ reproduced *kismet*^*10D26*^ phenotypes (Figures S1C, S1D, S1O, S1Q, S1S, S1U, and S1V). No phenotype was observed outside of clones, arguing against dominant-negative action of truncated proteins (Figures 1C, S1D, S1O, S1Q, and S1S). *kismet*^*10D26*^ phenotypes were rescued by a transgenic BAC construct (*kis*_*locus*_) encompassing the genomic locus containing *kismet* fused to a FLAP tag-encoding sequence (STAR Methods; Figures S1C–S1F). In addition, the expression of *kismet-L* cDNA, rescued clone size and increased stem cell number phenotypes of *kismet*^*10D26*^, *kismet*^*EC1*^, *kismet*^*LM27*^, and *kismet*^*1*^ alleles (Figures S1G, S1H, and S1M–S1V). In contrast, *kismet-S* cDNA expression did not rescue *kismet*^*10D26*^ phenotypes (Figures S1I, S1J, S1M, and S1N). Therefore, we conclude that *kismet-L* is required for normal midgut homeostasis. We will henceforth refer to *kismet-L* as “*kismet*.” Interestingly, the overexpression of an ATPase-dead version (Kismet^K2060R^) also showed a partial rescue of the mutant phenotype (Figures S1K–S1N). These data suggest that additional functional domains of Kismet are important for stem cell regulation, possibly by bridging interactions with other factors.

We then asked whether terminal differentiation was affected by the loss of *kismet*. *kismet* mutant clones, like wild-type clones, were able to produce terminally differentiated EC (Pdm1) and EE cells (Pros), though they made more per clone, consistent with larger clone sizes (Figures 1G–1P). Additional markers for differentiated EEs (peptide hormones DH31 and LTK2) and ECs (apical brush border) were detected in *kismet* mutants (Figures 1G–1J′, 1Q, and 1R). Thus, we conclude that loss of *kismet* function in ISCs results in increased stem cell numbers, without major defects in terminal differentiation.

### Kismet Activity Limits Proliferation and Self-Renewal of the ISC

**Figure 2:**
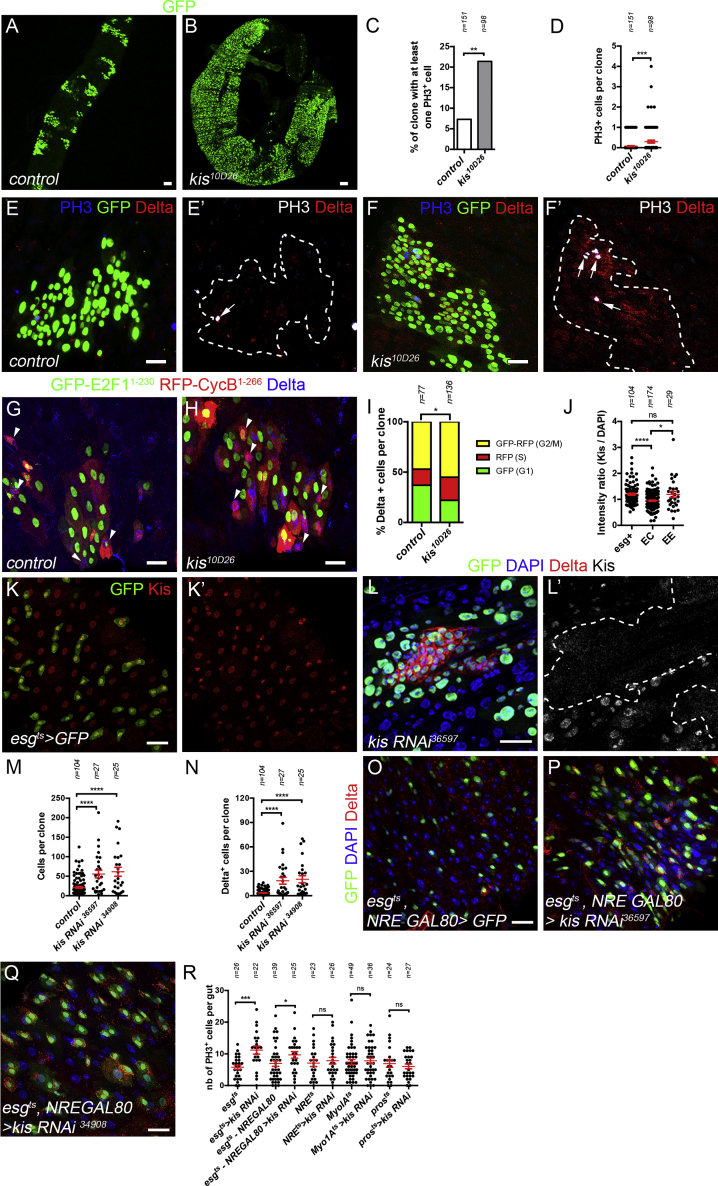
Loss of *kismet* Activity Promotes ISC Proliferation (A and B) Wild-type (A) and *kis*^*10D26*^ (B), MARCM clones at 30 days AHS. (C and D) Quantification of the percent of clones with at least one PH3+ cell (C) and the average number of PH3+ cell per clone (D) from (E)–(F′). (E–F′) Wild-type (E and E′), and *kis*^*10D26*^ (F and F′), 9 days AHS MARCM clones (arrows show PH3+ cells). (G and H) Wild-type (G) and *kis*^*10D26*^ (H), MARCM clones 9 days AHS expressing UAS-GFP-E2f^1-230^, UAS-mRFP-CycB^1-266^ FUCCI system allowing cell cycle stage determination (G1, nuclear GFP+; S: RFP+; and G2/M, GFP+ and RFP+; arrowheads show Delta+ ISCs). (I) Quantification of the percent ISCs (from G and H). (J) Mean Kismet fluorescence intensity normalized by mean DAPI staining in ISCs (esg+), EEs (diploid esg−), and ECs (polyploid cells) from (K) and (K′). (K and K′) Kismet showed ubiquitous nuclear expression with a stronger accumulation in esg+ progenitor cells (ISCs and EBs) marked by GFP and EE cells (diploid GFP−). (L and L′) *kismet* RNAi-expressing clones, 9 days AHS, had depleted Kismet protein and reproduced *kis*^*10D26*^ phenotypes. (M and N) Quantification of the number of cells per clone (M) and ISCs (Delta+) per clone (N) in wild-type and *kis RNAis* expressing clones. (O–Q) ISC-specific expression of GFP alone (O) or with *kis RNAi* BL36597 (P) or with *kis RNAi* BL34908 (Q) for 10 days at 29°C using *esg^ts^- NRE-GAL80* driver. (R) Quantification of the number of PH3+ cells per posterior midgut expressing *kis* RNAi in the ISCs and EBs (*esg*^*ts*^ driver), in ISCs only (*esg*^*ts*^*- NRE-GAL80* driver), in EBs only (*NRE*^*ts*^), in ECs only (*Myo1A*^*ts*^) for 10 days, or in EE cells (*pros*^*ts*^) for 10 days. A Fisher’s exact test was used in (C). A two-tailed Mann-Whitney test was used in (D), (J), (M), (N), and (R). A χ^2^ test was used for (I). Mean values in red; error bars, SEM; ns = non-significant, *p < 0.05, **p < 0.01, ***p < 0.001, ****p < 0.0001. Scale bars, 20 μm.

We then hypothesized that an increase in ISC numbers could be due to altered proliferation. Consistent with this, at 30 days after heat shock (AHS), *kismet* mutant tissue took over most of the midgut (Figures 2A and 2B), suggesting that mutant clones have a growth advantage over both heterozygous and wild-type cells.

Since ISCs are the primary dividing cell type in the midgut, we then assessed ISC proliferation using phospho-Histone H3 (PH3) as a marker for mitotic cells. 7.6% of the control clones had at least one and never more than one mitotic cell per clone (Figures 2C–2E′). In contrast, 21.4% of *kismet* mutant clones had at least one mitotic cell, and clones with more than one dividing cell were detected (Figures 2C, 2D, 2F, and 2F′). We assessed cell cycle parameters using the Fly-FUCCI system (Zielke et al., 2014) and found that *kismet* mutants had an increased proportion of ISCs in S phase and G2/early M at the expense of the G1 fraction (Figures 2G–2I). Together, these data further support the idea that *kismet* mutant ISCs have deregulated proliferation.

### Kismet Controls Cell Division in a Stem-Cell-Autonomous Manner

To determine in which cell *kismet* activity is required, we first assessed its expression profile in the intestine. An antibody recognizing the C-terminal part shared by the short and long forms of Kismet showed enrichment in cells positive for Escargot (esg), a marker of ISCs and EBs, and in EE cells compared to ECs (Figures 2J–2K′). To test cell-type-specific requirements of *kismet*, we verified that expressing *kismet RNAi* constructs mimicked *kismet* mutant phenotypes and led to the loss of Kismet protein (Figures 2L–2N and S2A–S2E′). ISC-specific or ISC and EB simultaneous knockdown of *kismet* led to an accumulation of ISCs and increased proliferation (Figures 2O–2R, S2F, and S2G). However, *kismet* knockdown in EEs, ECs, or in EB cells only did not show ISC phenotypes (Figures 2R and S2H–S2M). We conclude that *kismet* activity is required in ISCs to limit their proliferation to a basal level and to prevent abnormal expansion of their pool.

### Kismet Mutant Cells Activate Notch Signaling and Can Differentiate upon Forced Notch Activation

We next explored a potential impact of *kismet* on the Notch signaling pathway, which limits stem cell numbers by controlling daughter-cell-fate decisions (Bardin et al., 2010, Micchelli and Perrimon, 2006, Ohlstein and Spradling, 2006). We reasoned that as a regulator of transcription, Kismet might be necessary for expression of *Notch*. This was not the case, and Notch-expressing cells were in fact more abundant because of the accumulation of ISCs (Figures S3A and S3B). Furthermore, a reporter for the Notch pathway (NRE-lacZ; Notch Responsive Element), which is restricted to the EC-committed EB cells in wild-type tissue, was still expressed in *kismet* mutant clones, though absent from the extra Delta+ cells (Figures S3C–S3F).

We further examined whether *kismet* mutants might block Notch target gene activation. To test this, we induced *kismet* mutant clones and allowed 10 days of growth to accumulate extra ISCs (Figure S3G). We then induced expression of an active form of Notch in the mutant cells (N^Act^) by shifting to 29°C to inactivate a temperature-sensitive GAL80 (GAL80^ts^). At 18°C, control guts showed isolated Delta+ ISCs, whereas those with induced *kismet* mutant clones showed clusters of Delta+ ISCs (Figures S3H and S3I). However, expression of N^Act^ in *kismet* mutant clones that had accumulated ISCs caused differentiation of ISCs into ECs (Figures S3J, S3K, and S3L). Therefore, we conclude that *kismet* inactivation leads to the accumulation of extra-ISCs that maintain their potential to differentiate and activate Notch, though alteration in the kinetics of Notch signaling activation could not be excluded.

### Kismet Is Required to Maintain a Basal Level of Activation of EGFR Signaling

**Figure 3:**
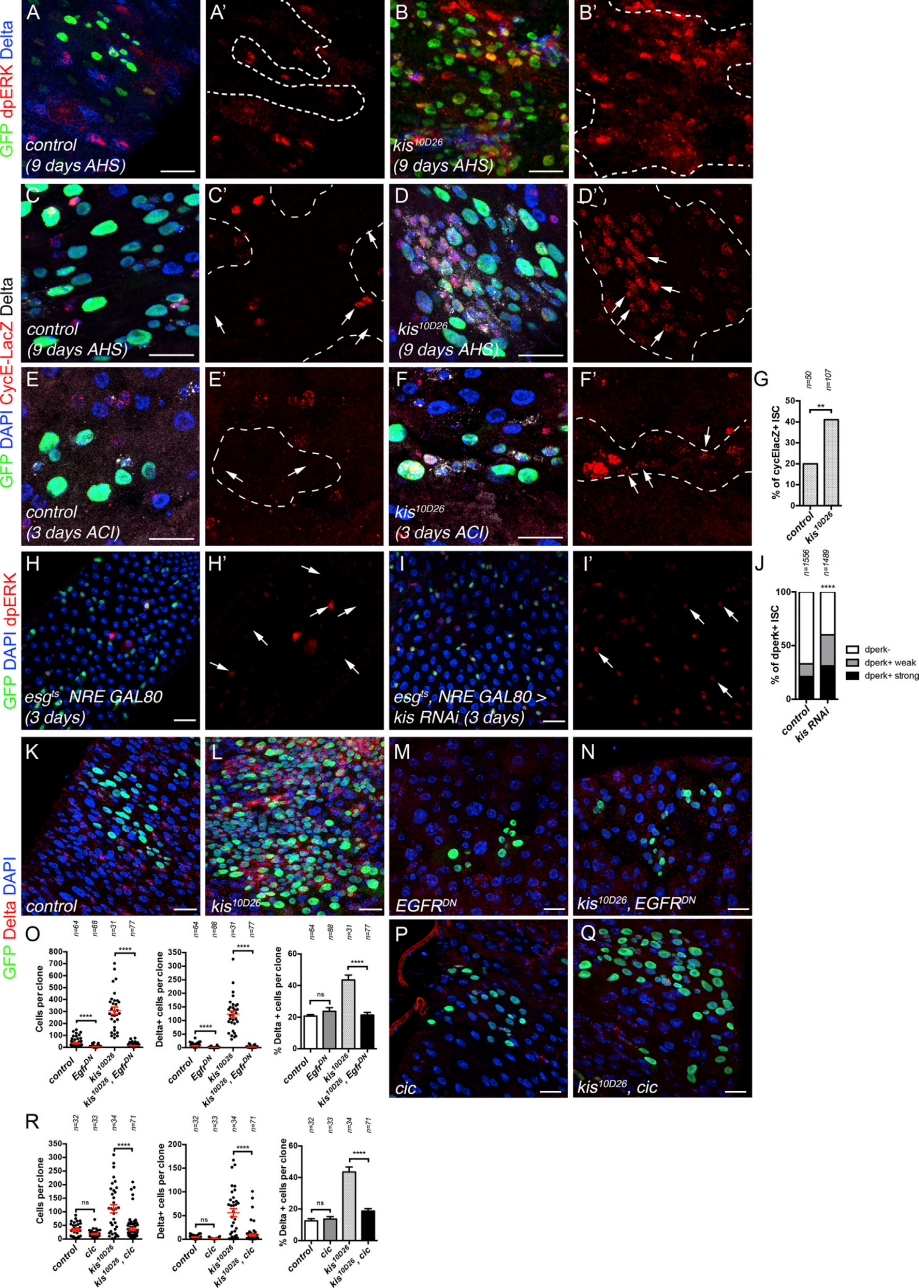
Kismet Controls Proliferation by Regulating EGFR Pathway Activity (A–D′) Wild-type (A, A′, C, and C′) and *kis*^*10D26*^ (B, B′, D, and D′), 9 days AHS MARCM clones. EGFR signaling, detected by dpERK (A–B′) and EGFR target CycE-LacZ (C–D′), was increased in *kis*^*10D26*^ clones. (E–F′) Wild-type (E and E′), and *kis*^*10D26*^ (F and F′), 3 days AHS MARCM clones expressing CycE-LacZ. (G) Proportion of ISCs expressing CycE-lacZ from (E) and (F′). (H and I) ISC-specific expression of GFP (H) or *kis RNAi* (BL34908) (I), 3 days at 29°C. (J) Quantification of proportion of ISCs showing strong, weak, or no dpERK from (H)–(I′). (K–N) 12-day AHS clones: wild-type (K), *kis*^*10D26*^ mutant (L), expressing *UAS-EGFR*^*DN*^ (M), and *kis*^*10D26*^ mutant expressing *UAS-EGFR*^*DN*^ (N). (O) Clone size, number of Delta+ cells/clone, and % of Delta+ cells/clone from (K)–(N). (P and Q) 10-day AHS clones: wild-type expressing *cic* (P) and *kis*^*10D26*^ mutant expressing *cic* (Q). (R) Clone size, number of Delta+ cells/clone, and % of Delta+ cells/clone from (P) and (Q). Results compared using a chi-square test in (G) and (J) and a two-tailed Mann-Whitney test in (O) and (R). Mean values in red; error bars, SEM; ns = non-significant, **p < 0.01, ****p > 0.0001. Arrows highlight ISCs. Scale bars, 20 μm.

As EGFR signaling is one of the primary signaling pathways controlling ISC proliferation (Biteau and Jasper, 2011, Buchon et al., 2010, Jiang et al., 2011), we then investigated whether *kismet* mutant stem cells may have increased EGFR signaling. In wild-type clones at 10 days AHS, a marker for activation of the EGFR pathway, dpERK, was weak in ISCs and mostly absent in other clonal cells (Figures 3A and 3A′). In *kismet* mutant clones, dpERK was strongly induced in ISCs and other cells of the clone (Figures 3B and 3B′). In addition, a reporter of *cyclin E*, acting downstream of EGFR signaling to regulate proliferation, was more highly induced in *kismet* mutant clones compared to wild-type at 9 days AHS and as early as 3 days AHS (Figures 3C–3G). Consistent with the rapid activation of EGFR signaling in *kismet* mutants, an increased proportion of ISCs with dpERK signaling was detected as early as 3 days after *kismet* RNAi induction in ISCs (Figures 3H–3J). Furthermore, the abnormal self-renewal of ISCs induced by the loss of *kismet* was abolished upon blocking EGFR signaling using a dominant negative form of EGFR (EGFR^DN^) or expression of *capicua* (*cic*), a downstream repressor of EGFR target genes (Figures 3K–3R). Not only was *kismet* mutant clone size reduced when EGFR signaling was downregulated, but there was also a reduction in the percent of ISCs per clone, returning to wild type (Figures 3O and 3R). This suggests that ectopic activation of EGFR signaling in *kismet* mutant clones drives extra cell division and promotes the accumulation of stem cells.

Previous work has shown that additional stress signaling pathways can further stimulate ISC proliferation due to feedback on EGFR signaling (Patel et al., 2015). In addition to EGFR signaling, Jun kinase signaling (assayed by puc-LacZ) and Jak/Stat signaling (assayed by 10XSTAT GFP) were also activated in ISCs of 9 days *kismet* mutant clones (Figures S4A–S4D′). The genetic inactivation of Yki, Stat, Insulin, and Jun kinase also reduced ISC proportion in *kismet* mutants (Figure S4E). In order to determine whether these pathways may also act early with EGFR signaling in *kismet* mutant stem cells to drive ISC proliferation, we examined a 3-day time point after clone induction. We did not detect early activation of the Jak/Stat ligands, Upd-LacZ, or the Jun Kinase reporter puc-LacZ in ISCs or Upd3-LacZ in ECs (Figures S4F–S4N). This argues against early activation of Jak/Stat and Jun Kinase pathways being initiating defects in *kismet* mutant responsible for ISC proliferation. Therefore, our data suggest that an initial enhancement of EGFR signaling occurs, followed by activation of additional pathways during the following 10 days of clone growth and further driving mutant clone growth. We conclude that Kismet is required to limit EGFR signaling in ISCs.

### Kismet Localizes to Chromatin Enriched in RNA Pol II and Brahma and Depleted for HP1, Histone H1, and Polycomb

**Figure 4:**
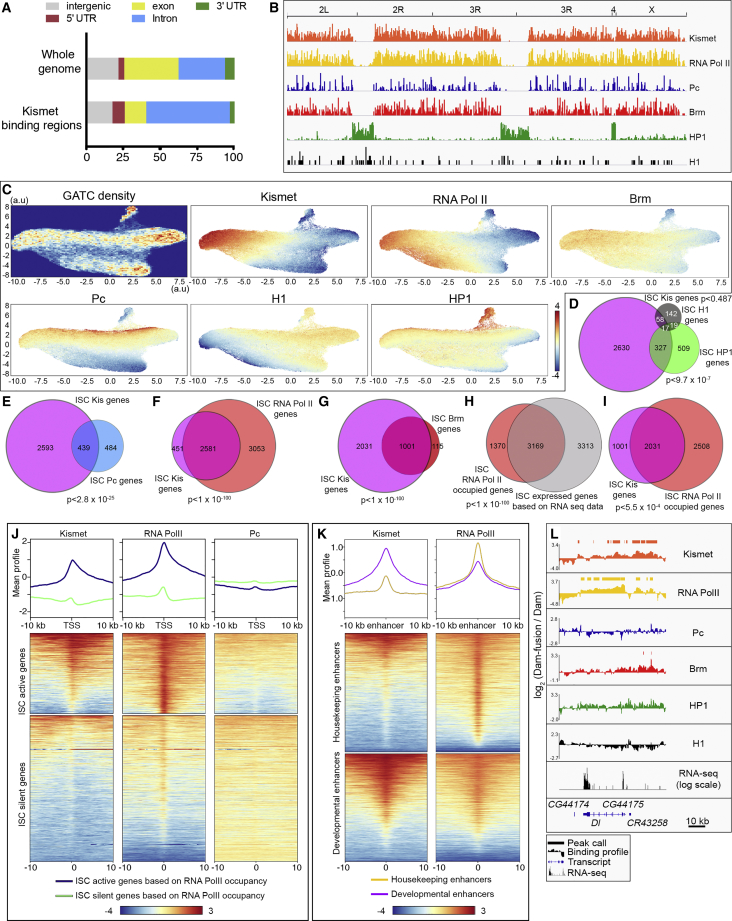
Genome-wide Mapping of Kismet Relative to RNA Pol II, Brm, Pc, HP1, and H1 (A) Kismet DamID-seq showed an enrichment of methylated GATCs in the introns and 5′UTRs of genes. Unassembled regions of the genome were not considered. (B) Genome-wide overview of the DamID binding peak density in ISCs of Kismet, RNA Pol II, Polycomb (Pc), Brahma (Brm), HP1, and H1. (C) UMAP clustering of GATC sites based on 7 DamID fusion proteins (see STAR Methods) in the ISC. Density of GATC sites throughout the genome used for clustering followed by the plots representing the binding of each protein over GATC sites. (D–I) Venn diagrams of genes with peaks of the DamID-seq data in ISCs: Kismet versus HP1 or H1 (D) versus Pc (E) versus RNA Pol II (F) and versus Brm (G). Genes with a significant mean RNA Pol II occupancy determined by DamID versus transcriptionally active genes based on RNA-seq from Dutta et al., 2015 (H) and versus genes with peaks of Kismet (I). (J) Mean position and metaplot of Kismet, RNA Pol II, and Pc in ISCs relative to the TSS for genes classified by their activity based on RNA Pol II occupancy. Kismet was significantly enriched over the TSS of active genes. (K) Mean position and metaplot of Kismet and RNA Pol II in ISC relative to previously defined “developmental” or “housekeeping” enhancers in S2 cells from Zabidi et al., 2015. Kismet was found enriched over developmental enhancers. (L) Wild-type RNA-seq, Dam-Kis, Dam-RNA Pol II, Dam-Pc, Dam-Brm, Dam-HP1, and Dam-H1 ISC binding profiles and peaks alignments over the genomic region surrounding the ISC-specific gene *Delta*.

To further understand how Kismet regulates ISC self-renewal, we identified Kismet-bound regions of the ISC genome using a targeted DamID-seq strategy (STAR Methods) (Marshall et al., 2016). A Dam-Kismet (Dam-Kis) fusion protein construct was expressed in ISCs using *esg*^*ts*^, *NRE-GAL80* for 1 day and significantly methylated GATC sites as compared to Dam-alone control expression were determined. Kismet distribution revealed enrichment in introns and 5′ UTRs (Figure 4A). We identified 3,032 genes containing Kismet peaks, defined by two consecutive significant GATC sites (p < 0.01) (Table S1).

To gain further insight into the type of chromatin bound by Kismet, we took advantage of DamID lines mapping different active and inactive chromatin states. Active regions are rich in RNA Pol II and Brahma, whereas inactive states are enriched in Polycomb (a reader of Histone H3K27me3), Histone H1, or HP1 (a reader of histone H3K9me3) (Marshall and Brand, 2017). Global patterns of Histone H1, HP1, and Polycomb binding were consistent with localization to repressed regions of the genome, whereas Brahma and RNA Pol II along with Kismet were enriched in active genic regions (Figures 4B and 4L). Histone H1 and HP1 were largely excluded from genic regions with HP1 strongly enriched at centromere-proximal regions of the genome and the 4^th^ chromosome, which is largely heterochromatic in flies (Figures S5H and 4B). These data are fully consistent with data from the nervous system (Marshall and Brand, 2017). We then examined global distribution of Kismet relative to RNA Pol II, Brahma, Polycomb, Histone H1, and HP1 using uniform manifold approximation and projection for dimension reduction (UMAP; Figure 4C [McInnes et al., 2018]). This approach allows the visualization and separation in a two-dimensional space, similar to a t-SNE (t-distributed stochastic neighbor embedding) plot, of GATC sites based on methylation levels by each of these factors (Figure 4C, GATC density map). Globally, Kismet had little overlap on UMAP with Histone H1 and HP1 (Figure 4C) or with genes containing peaks of Histone H1 and HP1 (2.5% and 11.3%, respectively; Figure 4D; Table S2). A majority of Kismet-rich GATC sites did not have strong Polycomb enrichment (Figure 4C; Table S2) and 14.5% of genes with Kismet peaks also had peaks of Polycomb (Figure 4E). Consistent with this, super-resolution imaging in polyploid EC cells, which allow better spatial resolution than ISCs, showed that Kismet and Histone H3K27me3 poorly colocalized (Figures S5A–S5A″). Therefore, we conclude that Kismet does not substantially localize to repressed chromatin domains.

Our data suggested, however, that Kismet more strongly overlapped with activated chromatin states, visualized on UMAP by Kismet enrichment with RNA Pol II and also to some extent with Brahma, a SWI/SNF chromatin remodeling factor associated with histone H3K27ac (Figure 4C). 85.1% of genes with peaks for Kismet also had peaks for RNA Pol II and 33.0% had a peak for Brahma (Figures 4F and 4G; Table S2). Together, these data suggest that Kismet localizes to transcriptionally active regions of the genome while being depleted in repressed chromatin.

### Kismet Localizes to Transcriptionally Active Genomic Regions and Developmental Enhancers

We then assessed how Kismet localized relative to active genes and enhancers. We established a list of expressed or “active” ISC genes based on Dam-RNA Pol II gene occupancy, a good proxy for gene expression (Marshall and Brand, 2015, Southall et al., 2013) (STAR Methods). 4,539 genes had significant occupancy suggesting that they are expressed, including many known ISC-enriched genes (*esg, sox21a, Delta,* and *spdo*; Table S3). Accordingly, these genes strongly overlap with published RNA sequencing (RNA-seq) data for ISC express genes (Dutta et al., 2015) (Figure 4H). We found that 67.0% of the genes with Kismet peaks were also occupied by RNA Pol II (Figure 4I). Genes enriched for Kismet and RNA Pol II or enriched for one but not the other were also detected (examples in Figures S5B–S5H). Separately analyzing ISC genes that were active (Dam-RNA Pol II occupied) or silent (Dam-RNA Pol II not occupied) revealed that Kismet, like its mammalian homologs CHD7/CHD8, was preferentially enriched around the transcription start sites (TSSs) of active genes (Figure 4J) (Schnetz et al., 2009, Schnetz et al., 2010).

Interestingly, while Kismet showed a strong enrichment for active genes (Figure 4J), it was also associated with a subset of silent genes in ISCs that are expressed in differentiated EE (Pros) and EC cells (Pdm1): 163 of 569 EE+ EC specifically enriched genes had Kismet peaks (from published RNA-seq (Dutta et al., 2015; see STAR Methods; examples in Figures S5C and S5D). Thus, while we detected no obvious roles in terminal differentiation, Kismet appeared to mark a subset of genes in ISCs that will be expressed during differentiation.

We then investigated the binding profiles of Kismet and RNA Pol II at previously published enhancers defined in S2 cells as being either “developmental” or “housekeeping” (Zabidi et al., 2015). Interestingly, Kismet showed enrichment over developmental enhancers, whereas RNA Pol II was enriched over both types of enhancers (Figure 4K). Thus, we conclude that Kismet is broadly distributed on many active genes in ISCs and is enriched at developmental enhancers.

### Knockdown of the Components of the Trr COMPASS-like Complex Mimic *kismet* Mutant Phenotypes

Kismet has previously been shown to restrict Histone H3K27me3 marks in the salivary gland (Dorighi and Tamkun, 2013, Srinivasan et al., 2008, Srinivasan et al., 2005). Therefore, we examined whether *kismet* inactivation in the gut had a similar effect on limiting Histone H3K27me3 but found no detectable global increase in H3K27me3 in *kismet* mutants (Figures S6A and S6B′). Previous studies have shown that Kismet acts in the salivary gland cells via recruitment of the histone methyltransferases Ash1 and Trx (Dorighi and Tamkun, 2013). Arguing against this possibility, we found that neither *ash1* nor *trx* clonal loss led to deregulation of ISC proliferation (Figures S6C–S6E, S6H, and S6I). Similarly, Brahma has been shown to co-localize with Kismet and share similar functions in transcription elongation (Armstrong et al., 2002, Srinivasan et al., 2005). However, consistent with previously published data in the intestine, *brahma*-RNAi-expressing clones were smaller and had fewer ISCs per clone than controls (Figures S6F, S6H, and S6I) (Jin et al., 2013). Therefore, our data suggest that Kismet mediates its action on ISC proliferation through direct or indirect interaction with additional chromatin regulators.

**Figure 5:**
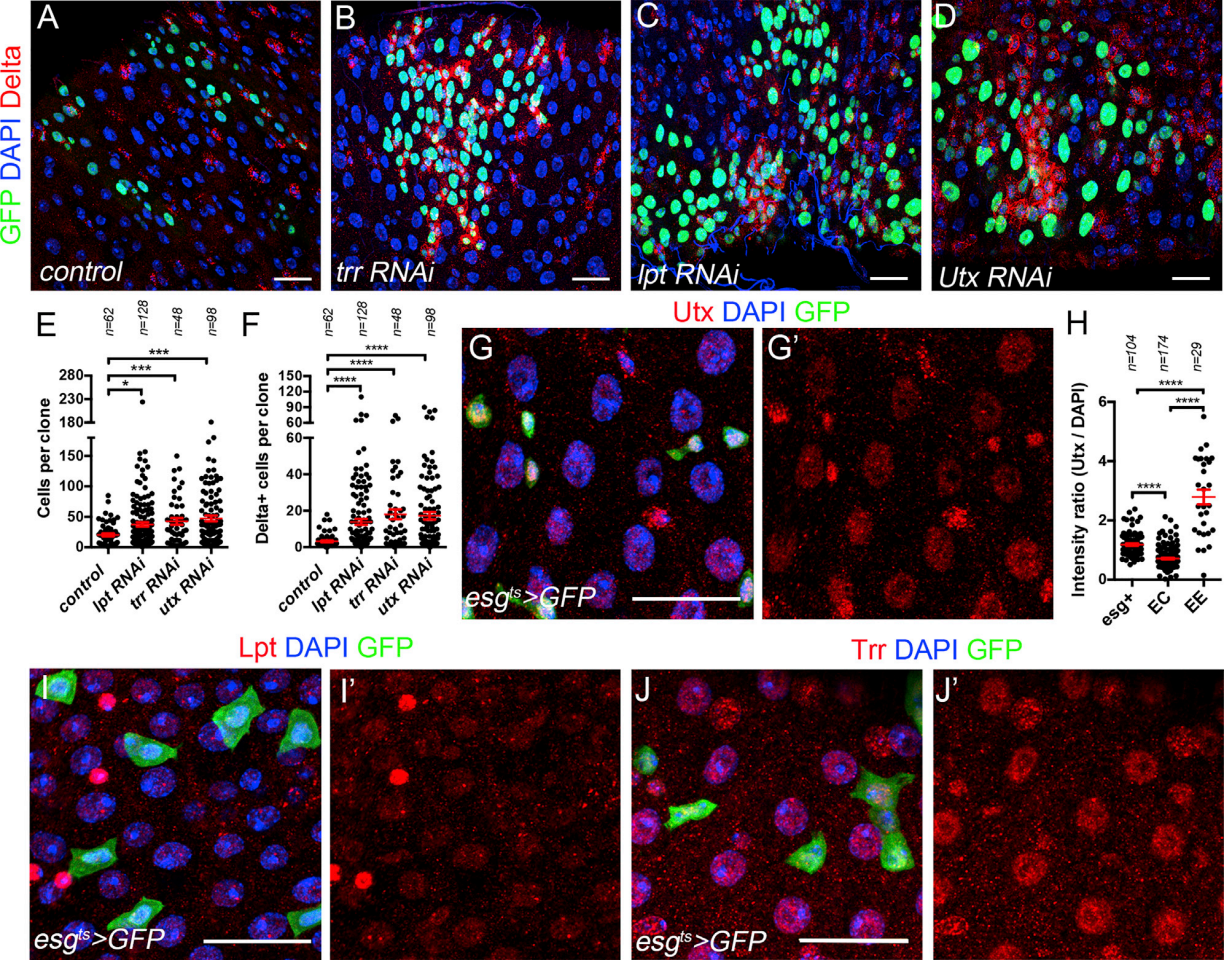
Loss of Trr COMPASS-like Complex Activity Induces Abnormal ISC Accumulation (A–D) Wild-type (A), *trr RNAi* (B)*, lpt RNAi* (C), or *Utx RNAi* (D) at 9 days AHS clones. (E and F) Average number of cells (E) and ISCs (F) per clone from (A)–(D). (G and G′) Utx was ubiquitously expressed but enriched in esg>GFP+ cells and in EE (esg>GFP− diploid cells). (H) Mean Utx staining fluorescence intensity normalized by the mean DAPI staining in ISCs, EEs, and ECs (polyploid cells) from (G) and (G′). (I and I′) Lpt was ubiquitously expressed but enriched in EE cells (diploid; esg>GFP−). (J and J′) Trr was uniformly expressed in all midgut cell types. In (E), (F), and (H), a two-tailed Mann-Whitney statistical test was used. Mean values in red; error bars, SEM; n = non-significant, *p < 0.05, ***p < 0.001, ****p < 0.0001. Scale bars, 20 μm.

We reasoned that additional Trithorax group genes might function with Kismet and sought to identify these factors. To this end, we tested the effect of the histone methyl transferases-encoding genes (Mohan et al., 2011): dSet1 (Set1A/Set1B in mammals), and Trr (MLL3/4 in mammals). The clonal expression of a previously validated RNAi construct against *set1* (Herz et al., 2012) had no obvious effects on ISCs or clone size (Figure S6G). However, knockdown of genes encoding Trr-complex proteins (Trr, Lost polyhomeotic domains of Trr [Lpt], and the histone demethylase Utx) revealed phenotypes similar to *kismet* (Figures 5A–5F). They had an increased number of cells per clone and Delta+ cells per clone (Figures 5A, 5B, 5E, and 5F). Clones of the *trr*^*B*^ mutant allele showed less severe but significant accumulation of extra-Delta+ diploid ISCs (Figures S6J–S6L′). Furthermore, the knockdown of *lpt* and *Utx* also exhibited *kismet*-mutant-like phenotypes (Figures 5C–5F). Analysis of Trr, Lpt, and Utx showed ubiquitous expression in the cell types of the midgut (Figures 5G–5J′). Utx, however, was enriched in the ISC and EB (esg+) progenitor cells and EEs similar to Kismet localization (Figures 5G–5H), and Lpt was enriched in EEs (Figures 5I and 5I′). The phenotypic similarity between *kismet* alleles and knockdown of *trr*-complex genes suggests they may collaborate to regulate ISC function. This idea is further supported by our findings that Kismet is enriched at developmental enhancers and that the Trr/MLL3/4 complexes have well-described functions in activating enhancers (Dorighi et al., 2017, Herz et al., 2012, Hu et al., 2013, Lee et al., 2013). Thus, we hypothesize that Kismet regulates ISC proliferation in conjunction with the Trr complex.

### Genome-wide Co-occupancy of Kismet and Trr and Co-regulation of Genes

**Figure 6:**
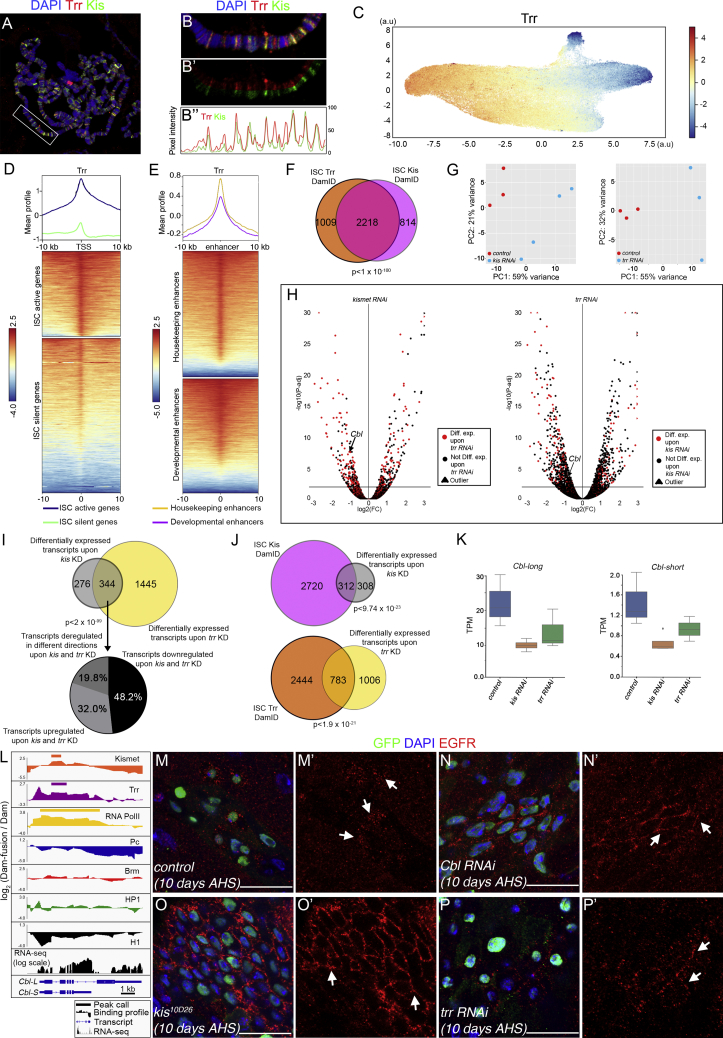
Genome-wide Mapping of Trr DNA Binding Sites and Genes Regulated by Kismet and Trr (A) Kismet and Trr localization on polytene chromosomes of the salivary gland. (B–B″) Magnification of the chromosome highlighted in (A). Fluorescent intensity in (B″). (C) Trr binding in the ISC clustered using UMAP based on 7 DamID fusion proteins (STAR Methods and Figure 4C). (D) Mean position and metaplot of Trr in ISCs plotted relative to the TSS for genes according to their activity based on RNA Pol II occupancy shows its enrichment over the TSS of active genes. (E) Mean position and metaplot of Trr in ISC over previously defined “developmental” or “housekeeping” enhancers in S2 cells from Zabidi et al., 2015. (F) Overlap between genes with peaks of Kismet versus Trr. (G) Principal-component analysis of RNA-seq. (H) Differentially expressed genes; red points highlight common genes. (I) Upper: overlap between genes with RNAs deregulated upon *kis* and *trr* knockdown in the ISCs. Lower: proportion of RNAs altered in *kis* and *trr* knockdown. (J) Upper: genes with peaks of Kismet and deregulated after *kis* knockdown in the ISCs. Lower: genes with peaks of Trr and deregulated after *trr* KD in the ISCs. (K) RNA-seq data showing downregulation of the 2 *Cbl* isoforms upon either *kis* RNAi and *trr* RNAi in the ISC. (L) Alignement at the *Cbl* locus of wild-type RNA-seq, Kismet, Trr, RNA Pol II, Pc, Brm, HP1, H1 binding profiles and peaks as determined by DamID-seq in ISCs. (M–P′) clone of wild-type (M and M′), *Cbl* RNAi (N and N′), *kis*^*10D26*^ (O and O′) and *trr RNAi* (P and P′), 10 days AHS. Arrows show EGFR-positive cells. Scale bars, 20 μm.

A prediction of collaboration between Kismet and Trr complex is that they may co-bind and co-regulate genes. We found that the distribution of Kismet on polytene chromosomes largely overlapped with Trr (Figures 6A–6B″) and in ISCs using targeted DamID-seq of Trr (Figure 6C). Like Kismet, Trr was enriched over the TSS of active genes as compared to inactive genes (based on RNA Pol II occupancy; Figure 6D). Interestingly, while Kismet had preferential enrichment for developmental enhancers (Figure 4K), Trr was equally enriched at both developmental and housekeeping enhancers (Figure 6E). Examining peaks in genes, 73.2% of Kismet-bound genes were co-bound by Trr (Figure 6F; Table S4). We conclude that Kismet and Trr share genome-wide localization and binding to the genes, supporting our hypothesis that they act in concert.

We then wanted to assess whether similar genes might be deregulated upon *kismet* or *trr* knockdown. To this end, we used fluorescence-activated cell sorting (FACS) to sort adult ISCs (*esg*^*ts*^>*GFP*; *NRE-GAL80*) of controls (white RNAi) or those expressing RNAi against *kismet* or *trr* and performed RNA-seq (Figures 6G–6I; Table S5). Interestingly, 50.3% of genes having altered RNA upon *kismet* knockdown and 43.8% of genes having altered RNA levels upon *trr* knockdown showed Kismet and Trr binding by DamID-seq, respectively (Figure 6J). This suggests that these genes are directly regulated by Kismet and Trr. However, the deregulated genes represented only 10.3% and 24.3% of the total Kismet and Trr-bound genes, respectively. Therefore, the genetic perturbation of relatively general chromatin binding factors can affect a limited subset of genes. This likely underlies the specific phenotypes of general chromatin factors noted both here in ISCs and in other contexts, such as during human development (Bajpai et al., 2010, Schulz et al., 2014b, Vissers et al., 2004).

Importantly, we found that 55.5% of the RNAs altered upon knockdown of *kismet* were also altered in *trr* knockdown (Figure 6I). This represented 19.2% of the *trr*-altered transcripts. Among these genes deregulated upon both *kismet* and *trr* knockdowns, 80.2% are in the same direction, with a decrease in transcript level (Figure 6I).

Overall, these data strongly support our hypothesis that Kismet and Trr co-bind throughout the genome and co-regulate gene expression.

### Kismet and Trr Promote Expression of the E3 Ligase Cbl to Maintain Low Levels of the EGF Receptor

Our previous data indicated that *kismet* mutant ISCs have increased EGFR signaling (Figures 3A–3G). Similarly, upon *trr* knockdown, we found deregulation of dpERK (Figures S7A–S7B′). The similarities in loss-of-function phenotypes of *kismet* and *trr*, the colocalization in the genome, the co-regulation of many genes, and deregulation of EGFR signaling led us to propose that Kismet and the Trr complex act together to limit ISC self-renewal through EGFR regulation.

**Figure 7:**
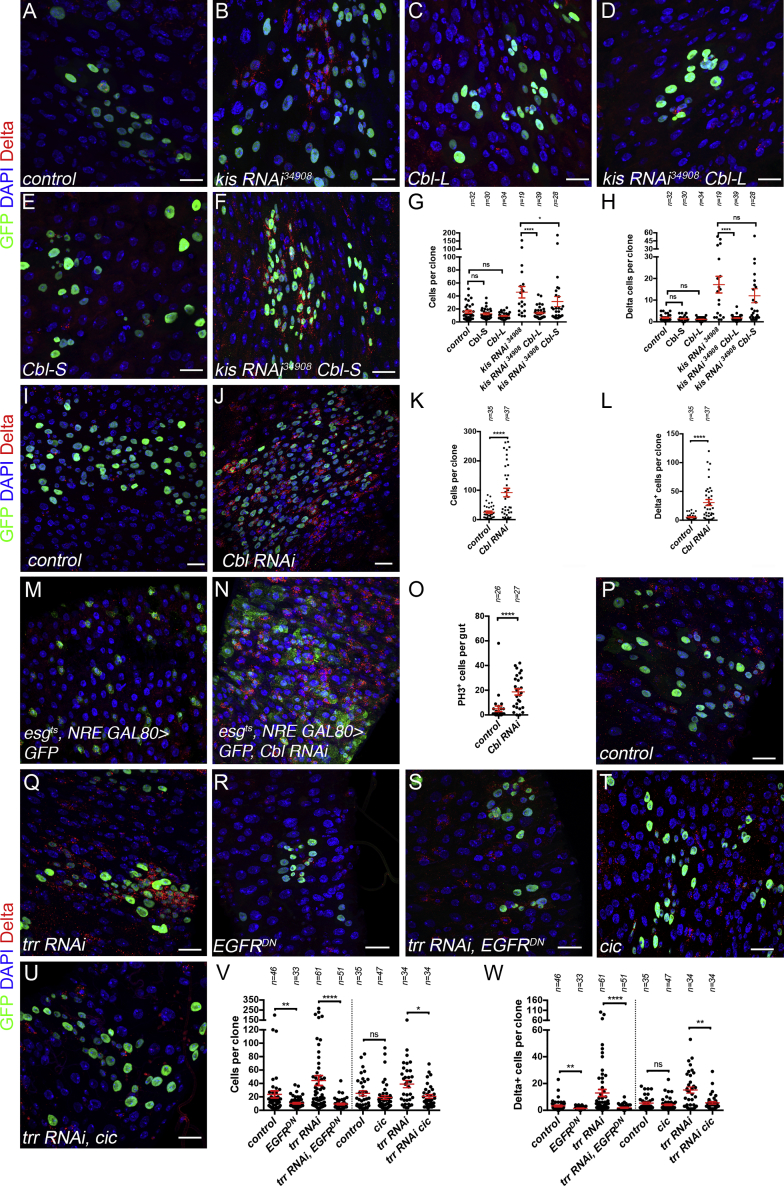
Trr and Kismet Regulates EGFR Activity through the Control of *Cbl* Expression (A–F) Clone of wild-type (A), *kis RNAi*^*34908*^ (B), *UAS-Cbl-L* (C), both *kis RNAi*^*34908*^ and *UAS-Cbl-L* (D), *UAS-Cbl-S* (E), and both *kis RNAi*^*34908*^ and *UAS-Cbl-S* (F) at 9 days AHS. (G and H) Average number of cells and ISC per clone from (A)–(F). (I and J) Clones of wild-type (I) and *Cbl* RNAi (J) 10 days AHS. (K and L) Average number of cells and ISC per clone from (I) and (J). (M and N) ISC-specific expression of GFP (M) and *Cbl RNAi* (N) driven by *esg*^*ts*^*- NRE-GAL80* for 10 days at 29°C. *Cbl* knockdown results in an accumulation of GFP, Delta+ cells. (O) Quantification of number of PH3+ cells per gut from (M) and (N). (P–U) Clone of wild-type (P), *trr RNAi* (Q), *UAS-EGFR*^*DN*^ (R), both *trr RNAi* and *UAS-EGFR*^*DN*^ (S), *UAS-cic* (T), and both *trr RNAi* and *UAS-cic* (U) at 10 days AHS. (V and W) Clone size (V) and number of Delta+ per clone (W) from (P)–(U). Results were compared using a two-tailed Mann-Whitney statistical test. Mean values in red; error bars, SEM; n = non-significant, *p < 0.05, **p < 0.01, ****p < 0.0001. Scale bars, 20 μm.

Assessing our DamID-seq and RNA-seq data for regulators of EGFR signaling, we identified *Cbl*, encoding an E3 ligase known to promote degradation of EGFR (Duan et al., 2003, Hime et al., 1997, Levkowitz et al., 1999, Meisner et al., 1997, Pai et al., 2000, Soubeyran et al., 2002). *Cbl* was bound by both Kismet and Trr and, long and short isoforms of *Cbl* were downregulated upon inactivation of *kismet* and *trr* (Figures 6H, 6K, and 6L). Of note, none of the core components of the Hippo pathway are deregulated upon *kismet* knockdown, arguing against this pathway being involved in initiation of *kismet* mutant phenotypes. If Kismet and Trr control the levels of EGFR via Cbl, then *kismet* and *trr* knockdown contexts would lead to increased EGFR protein. Clones expressing RNAi against *Cbl*, *kismet*, or mutant for *kismet*, had a strong increase in EGFR levels, both in clones at 10 days AHS and upon 3-day induction in ISCs (Figures 6M–6O′, S6O–S6R′, and S6T). Knockdown of *trr* also showed increased EGFR levels in 10 days clones (Figures 6P and 6P′), although upon 3-day knockdown in ISC, this was less pronounced than that of *kismet* knockdown (Figures S6Q, S6S, and S6T), consistent with *Cbl* transcripts being less reduced upon *trr* knockdown than upon *kismet* knockdown (Figure 6K). Furthermore, the expression of *Cbl-L* isoform (but not *Cbl-S*) in *kismet* RNAi-expressing clones rescued clone size and accumulation of Delta+ cells supporting that *Cbl* acts downstream of Kismet to regulate ISC self-renewal (Figures 7A–7H). In agreement with previous work (Jiang et al., 2011), the knockdown of *Cbl* led to larger clones with increased numbers of Dl+ ISCs and excessive proliferation (Figures 7I–7O). In addition, *trr* mutant phenotypes could be suppressed by conditions that lowered EGFR signaling (expression of EGFR-DN or *cic*; Figures 7P–7W).

Finally, if *kismet* and *trr* act on similar target genes, we would predict that their combined phenotype would be like *kismet* mutants, which is indeed what was found (Figures S7C–S7G). Together, our data suggest that one of the downstream targets of Kismet and Trr required to maintain a basal level of ISC proliferation is the E3 ligase encoding gene *Cbl*, which modulates EGFR protein levels and signaling activation.

## Discussion

Through an unbiased genetic screen, we have identified the chromatin remodeling factor Kismet/CHD7/CHD8, as a regulator of stem cell proliferation in the fly intestine. By establishing the first genome-wide binding map of Kismet in *Drosophila*, our data revealed a large overlap with transcriptionally active regions. Interestingly, our genome-wide mapping and RNA-seq data suggest that Kismet mediates its role on ISCs through collaboration with the Trr complex and that the negative regulator of EGFR, *Cbl*, is one critical downstream direct target of *kismet* and *trr*, leading to the deregulation of EGFR signaling. Altogether, our data uncover an important level of chromatin regulation required to dampen the ISC proliferative response in routine homeostasis.

In response to homeostatic cell turnover and induced damage, many signaling pathways regulate ISC proliferation rates, though how chromatin regulation impinges on this was not understood. Our data show that Kismet and Trr play essential roles in preventing excess ISC proliferation through limiting EGFR signaling, which may be required after stress for the return to basal levels of proliferation. In addition to EGFR signaling, we find that inhibition of additional pathways can suppress *kismet* phenotypes, reminiscent to “niche appropriation” described for *Notch* mutant tissue: after initial deregulation of stem cell proliferation and local tissue perturbation due to *Notch* inactivation, multiple signaling pathways become activated, including Jnk and Jak/Stat signaling, which function together to further drive ISC proliferation (Patel et al., 2015). Thus, our findings suggest that niche appropriation is a general property of rapidly proliferating tissues that create stress signals in the gut, further fueling cell division.

Interestingly, our findings suggested that Kismet is enriched at genes that are expressed not only in ISCs but also at a subset of genes that is OFF in the stem cell but will be later turned ON during the differentiation process. Of note, we did not detect obvious defects in terminal cell fate differentiation in *kismet* mutant clones using well-characterized markers of EE and EC cells. Further analysis of *kismet* mutant EE and EC cells will determine whether there are more subtle defects in differentiation or not. This raises the possibility that Kismet may be a good marker of pre-patterning of lineage-specific genes.

Our data suggest that Kismet cooperates with the Trr complex to regulate many genes. CHARGE syndrome, due to a heterozygous mutation of *CHD7* in humans, has extensive phenotypic overlap with Kabuki syndrome, caused by mutation of *MLL4* (also known as *KMT2D* and *MLL2*) and *UTX* (also known as *KDM6A*) (Butcher et al., 2017, Miyake et al., 2013, Ng et al., 2010, Schulz et al., 2014a). These data raise the question of a similar collaboration between these enzymes in human development. The Trr/MLL3/4 complexes establish the H3K4me1 mark, enriched at primed and active enhancers and, to some extent, promoters. Could Kismet/CHD7 remodeling activity promote H3K4me1? Our data argue against this since H3K4me1 was reduced in the gut upon knockdown of *trr* and *lpt* (Figures S7L–S7N′), but not altered in *kismet* mutant clones (Figures S7O and S7O′). In addition, no obvious defects were detected in histone H3K27ac in *kismet* mutant clones (Figures S6M–S6N′) or protein levels of Trr in *kismet* mutants or of Kismet in *trr* mutants were found (Figures S7H–S7K′). It is possible, however, that Kismet/CHD7 might promote the methyltransferase-independent activity of Trr/MLL3/4 that has been shown to regulate enhancer efficiency (Dorighi et al., 2017, Rickels et al., 2017). Further evidence suggesting a molecular link between Kismet/CHD7/CHD8 and the Trr/MLL3/4 complex comes from co-immunoprecipitation (coIP) between Kismet and CBP, a binding partner of the Trr complex component Utx and between CHD7/CHD8 and the MLL3/4 complex components WDR, ASH2L, and RbBP5 (Schulz et al., 2014a, Thompson et al., 2008, Tie et al., 2012). Thus, we speculate that Kismet and the Trr complex may both be necessary for regulation of a subset of genes, such as *Cbl*.

In addition to similar phenotypes of *CHD7* and *MLL4* mutations on human development, both *CHD7* and *MLL3*/*4* complex components are mutated in cancers. CHD7 is found as a highly expressed fusion protein in small cell lung cancers (Pleasance et al., 2010). A subset of colorectal carcinomas and gastric cancers were found to have frequent mutations in both *CHD7* and its closely related gene, *CHD8* (Kim et al., 2011, Sawada et al., 2013, Tahara et al., 2014). Mutations in *CHD7* and in the MLL3/4 complex are frequently found in medulloblastoma, and MLL3/4 is also found inactivated in many additional cancers (Ford and Dingwall, 2015, Robinson et al., 2012). How deregulating *CHD7* and *MLL3/4* may impact cancer progression is not entirely clear, though recent studies linking enhancer and super-enhancer deregulation to cancer formation suggest that the deregulation of enhancers upon mutation of Kismet/CHD7 and the Trr/MLL3/4 complex could drive aberrant proliferation (Herz et al., 2014, Hnisz et al., 2013). While much of what we know about chromatin regulation in stem cells comes from studies of cultured embryonic stem cells, our work provides insight into *in vivo* roles of chromatin remodeling factors in control of adult stem cell self-renewal and proliferation programs. Our findings that Kismet and the Trr complex loss lead to dramatic alteration of ISC proliferation indicate that the *Drosophila* intestine will be a useful model to probe the relationship between chromatin regulation and stem cell proliferation control.

## STAR★Methods

### Key Resources Table

**Table:** 

REAGENT or RESOURCE	SOURCE	IDENTIFIER
**Antibodies**		

Mouse anti-Delta ECD (1:2000)	DSHB	Cat# c594.9b; RRID: AB_528194
Mouse anti-Notch ECD (1:100)	DSHB	Cat# c458.2h; RRID: AB_528408
Mouse anti-Prospero (MR1A; 1:1000)	DSHB	RRID: AB_528440
Chicken anti-GFP (1:2000)	Abcam	Cat# ab13970; RRID: AB_300798
Goat anti-ßGAL (1:500)	Biogenesis	Cat# 466–1409
Rabbit anti-Pdm1 (1:1000)	Gift from X. Yang, Zhejiang University, China	RRID: AB_2570215
Rabbit anti-PH3 (1:1000) Millipore	Millipore	Cat# 06-570; RRID: AB_310177
Goat anti-Kismet DK20 (1:500)	Santa Cruz	sc-15848; RRID: AB_672122
Rabbit anti-Utx, (1:500)	Gift from A. Shilatifard, Northwestern University Feinberg School of Medicine	RRID: AB_2567973
Rabbit anti-Trr (1:500)	Gift from A. Shilatifard, Northwestern University Feinberg School of Medicine	RRID: AB_2568870
Rabbit anti-Lpt (1:500)	Gift from A. Shilatifard, Northwestern University Feinberg School of Medicine	RRID: AB_2568872
Rabbit anti-H3K4me1 (1:500)	Gift from A. Shilatifard, Northwestern University Feinberg School of Medicine	N/A
Rabbit anti-dpERK (1:200)	Cell Signaling Technology	Cat# 4377; RRID: AB_331775
Rabbit anti-H3K27me3 (1:500)	Diagenode	Cat# C15410195; RRID: AB_2753161
Rabbit anti-H3K27ac (1:2000)	Abcam	Cat# ab4729; RRID: AB_2118291
Mouse anti-EGFR (1:100)	Sigma	Cat# E2906; RRID: AB_609900
Rabbit anti-DH31 (1:500),	Gift from J.A. Veenstra, Université de Bordeaux	RRID: AB_2569126
Rabbit anti-LTK2 (1:1000)	Gift from J.A. Veenstra, Université de Bordeaux	N/A

**Chemicals, Peptides, and Recombinant Proteins**		

Alexa 647-conjugated phalloidin (1:100)	LifeTechnologies	Cat# A22287: RRID: AB_2620155
Quick ligase	NEB	Cat# M2200S
RNase A	Sigma	Cat# R6513-50MG
T4 DNA ligase	NEB	Cat# B0202S
DpnI	NEB	Cat#R0176L
DpnII	NEB	Cat#R0543L
Sau3AI	NEB	Cat# R0169L
PCR buffer MyTaq HS	Bioline	Cat# BIO-21111
AlwI	NEB	Cat# R0513S
Klenow fragment	NEB	Cat# 210S
T4 polynucleotide kinase	NEB	Cat# M0201S
Elastase	Sigma	Cat# E0258-5MG

**Critical Commercial Assays**		

Qiaquick PCR Purification kit	Qiagen	Cat# 28104
QIAmp DNA Micro Kit	Qiagen	Cat# 56304
Arcturus PicoPure RNA Isolation Kit	ThermoScientific	Cat# KIT0202
RNase-Free DNase Set	Qiagen	Cat# 79254
Arcturus™ RiboAmp™ HS PLUS Kit	ThermoScientific	Cat# KIT0525

**Deposited Data**		

Lists of expressed genes and cell type-specific genes were generated from published RNAseq data in the gut	Dutta et al., 2015	http://flygutseq.buchonlab.com/resources

**Experimental Models: Organisms/Strains**		

Drosophila: FRT40A kis10D26	This study, Institut Curie Paris.	N/A
Drosophila: UAS-LT3-NDam	Southall et al., 2013	N/A
Drosophila: kis^1^	Daubresse et al., 1999	Cat# 431, RRID:BDSC_431
Drosophila: UAS-kis-RNAi #36597	BDSC, (Perkins et al., 2015)	Cat# 36597, RRID:BDSC_36597
Drosophila: UAS-kismet RNAi #34908	BDSC, (Perkins et al., 2015)	BDSC Cat# 34908, RRID:BDSC_34908)
Drosophila: UAS-EGFR^DN^	BDSC (Buff et al., 1998)	BDSC Cat# 5364, RRID:BDSC_5364
Drosophila: UAS-lpt-RNAi	BDSC, (Perkins et al., 2015)	BDSC Cat# 25994, RRID:BDSC_25994
Drosophila: UAS-trr-RNAi	BDSC, (Perkins et al., 2015)	BDSC Cat# 29563, RRID:BDSC_29563
Drosophila: UAS-utx-RNAi	BDSC, (Perkins et al., 2015)	BDSC Cat# 34076, RRID:BDSC_34076
Drosophila: FR82B trx^E2^	BDSC (Gindhart and Kaufman, 1995)	BDSC Cat# 24160, RRID:BDSC_24160
Drosophila: FR19A trr^B^	BDSC (Haelterman et al., 2014)	BDSC Cat# 57138, RRID:BDSC_57138
Drosophila: UAS-bsk^DN^	BDSC (Adachi-Yamada et al., 1999)	BDSC Cat# 6409, RRID:BDSC_6409
Drosophila: UAS-yki-RNAi	BDSC, (Perkins et al., 2015)	BDSC Cat# 34067, RRID:BDSC_34067
Drosophila: UAS-InR^DN^	Gift to BDSC by Exelixis, Inc.	BDSC Cat# 8253, RRID:BDSC_8253
Drosophila: UAS-domeRNAi	BDSC, (Perkins et al., 2015)	BDSC Cat# 34618, RRID:BDSC_34618
Drosophila: UAS-ash1-RNAi # 31050	BDSC, (Perkins et al., 2015)	BDSC Cat# 31050, RRID:BDSC_31050
Drosophila: UAS-ash1-RNAi # 36130	BDSC, (Perkins et al., 2015)	BDSC Cat# 36130, RRID:BDSC_36130
Drosophila: UAS-brm-RNAi	BDSC, (Perkins et al., 2015)	BDSC Cat# 31712, RRID:BDSC_31712
Drosophila: UAS-Cbl-RNAi	BDSC, (Perkins et al., 2015)	BDSC Cat# 27500, RRID:BDSC_27500
Drosophila: UAS-GFP-E2f^1-230^, UAS-mRFP-CycB^1-266^	BDSC (Zielke et al., 2014)	BDSC Cat# 55118, RRID:BDSC_55118
Drosophila: 10XSTAT92E-GFP	BDSC (Ekas et al., 2006)	BDSC Cat# 26198, RRID:BDSC_26198
Drosophila: CycE-lacZ	BDSC Gift by Helena Richardson, Peter MacCallum Cancer Centre to BDSC	BDSC Cat# 30722, RRID:BDSC_30722
Drosophila: UAS-set1-RNAi	VDRC, (Dietzl et al., 2007)	Cat# 40682
Drosophila: UAS-cic-RNAi	VDRC, (Dietzl et al., 2007)	Cat# 103805
Drosophila: FRT40A kis^LM27^	Gift from D.R. Marenda, Drexel University.	N/A
Drosophila: FRT40A kis^EC1^	Gift from D.R. Marenda	N/A
Drosophila: NRE-LacZ	Furriols and Bray, 2001	N/A
Drosophila: UAS-Notch^cdc10^	Brennan et al., 1999	N/A
Drosophila: UAS-LT3-NDam-RNAPol II	Southall et al., 2013	N/A
Drosophila: UAS-LT3-Dam-Pc	Marshall and Brand, 2017	N/A
Drosophila: UAS-LT3-Dam-HP1a	Marshall and Brand, 2017	N/A
Drosophila: UAS-LT3-Dam-Brm	Marshall and Brand, 2017	N/A
Drosophila: UAS-LT3-Dam-H1	Marshall and Brand, 2017	N/A
Drosophila: UAS-cic^HA^	Jin et al., 2015	N/A
Drosophila: pucE69-LacZ	Gift from N.Tapon, Francis Crick Institute, London	N/A
Drosophila: Upd-LacZ	Gift from B.A. Edgar, Huntsman Cancer Institute, Utah	N/A
Drosophila: Upd3.1-LacZ	Gift from B.A. Edgar, Huntsman Cancer Institute, Utah	N/A
Drosophila: UAS-Cbl-L	Gift from L.M. Pai, Chang Gung University, Taiwan	N/A
Drosophila: UAS-Cbl-S	Gift from L.M. Pai, Chang Gung University, Taiwan	N/A
Drosophila: NRE-GAL4 ; tubGAL80^ts^ UAS-GFP	Zeng et al., 2010	N/A
Drosophila: esg-GAL4, tubGAL80^ts^ UAS-GFP	Jiang et al., 2009	N/A
Drosophila: esg-GAL4 UAS-GFP; Su(H)GBE-GAL80 tubGAL80^ts^	Wang et al., 2014	N/A
Drosophila: MyoIAGAL4; tubGAL80^ts^ UAS-GFP	Jiang et al., 2009	N/A
Drosophila: pros^voila^-Gal4, tub-Gal80ts	Balakireva et al., 1998	N/A
Drosophila: w P[hs-FLP] P[pTub-GAL4] P[UAS-nlsGFP]	Bardin et al., 2010	N/A
Drosophila: FRT40A P[pTub-GAL80]	Bardin et al., 2010	N/A
Drosophila: FRT82B P[pTub-GAL80]	Bardin et al., 2010	N/A
Drosophila: w P[hs-FLP]; FRT40A P[pTub-GAL80]; Drosophila: P[UAS-RFP], P[pTub-GAL4]	This paper, Institut Curie, Paris.	N/A
Drosophila: hsflp122 P[pTub-GAL80] FRT19A; P[pAct-GAL4] P[UASGFP]	Lin et al., 2008	N/A

**Recombinant DNA**		

Drosophila BAC: (P[acman] BAC CH322-128O7)	BACPAC Resources Center	CH322-128O7
Drosophila BAC: (P[acman] BAC CH321-35E09)	BACPAC Resources Center	CH321-35E9
Plasmid: kis_locus_-FLP	This paper, Institut Curie, Paris.	N/A
Plasmid: R6kam-hNGFP	Gift from A. A. Hyman, Max Planck Institute, Dresden.	N/A
Plasmid: UAS- KisS-Flag	This paper, Institut Curie, Paris.	N/A
Plasmid: UAS-KisL-His-Flag	This paper, Institut Curie, Paris.	N/A
Plasmid: UAS-kis-K2060R-His-Flag	This paper, Institut Curie, Paris.	N/A
Plasmid: pUASTattB-LT3-NDam	Southall et al., 2013	N/A
Plasmid: UAS-LT3-Dam-Kis	This paper, Institut Curie, Paris.	N/A
Plasmid: UAS-LT3-Dam-Trr	This paper, Institut Curie, Paris.	N/A

**Software and Algorithms**		

Prism 7	GraphPad Software	RRID:SCR_002798
FIJI	https://fiji.sc	N/A
Damidseq_pipeline	Marshall and Brand, 2015	https://owenjm.github.io/
bowtie2	Langmead and Salzberg, 2012	http://bowtie-bio.sourceforge.net/bowtie2/index.shtml
bedtools	Quinlan and Hall, 2010	https://bedtools.readthedocs.io/en/latest/index.html
DESeq2	Love et al., 2014	https://bioconductor.org/packages/release/bioc/html/DESeq2.html
deepTools plotHeatmap	Ramírez et al., 2014	https://deeptools.readthedocs.io/en/develop/content/tools/plotHeatmap.html
polii.gene.call	Marshall and Brand, 2015	https://owenjm.github.io/
Galaxy	Afgan et al., 2016	https://usegalaxy.org
IPython		https://ipython.org
Galaxy workflow	IPython notebooks and UMAP code used in this study - Institut Curie Paris	https://github.com/bardin-lab/kismet-analysis

### Contact for Reagent and Resource Sharing

Further information and requests for resources and reagents should be directed to and will be fulfilled by the Lead Contact, Allison Bardin (allison.bardin@curie.fr).

### Experimental Model and Subject Details

Flies were kept in yeasted tubes at 25°C unless mentioned. The following fly stocks were used in this study: *FRT40A kis*^*10D26*^ (from C.P., F.S., A.J.B., unpublished genetic screen), *kis*^*1*^ (#431)*, UAS-kis-RNAi* (*#36597*) for Figures 2L–2P, 2R, S2A–S2E′, S2G, S2I, S2K, and S2M and *UAS-kismet RNAi* #34908 for Figures 2M, 2N, 2Q, 3I, 3J, 7B, 7F, S6Q, S6R, and S6T and for *kismet* knockdown RNA-seq condition), *UAS-lpt-RNAi* (#25994), *UAS-trr-RNAi* (#29563), *UAS-utx-RNAi* (#34076), *FR82B trx*^*E2*^ (#24160), FRT19A *trr*^*B*^ encoding a putative truncated 512-aa protein *(#57138), UAS-EGFR*^*DN*^ (#5364), *UAS-bsk*^*DN*^ (#6409), *UAS-yki-RNAi* (#34067), *UAS-InR*^*DN*^ (#8253), *UAS-domeRNAi* (#34618), *UAS-ash1-RNAi* (#31050 and #36130), *UAS-brm-RNAi* (#31712), UAS-Cbl-RNAi (#27500), UAS-GFP-E2F^1-230^, UAS-mRFP-CycB^1-266^ (#55118), 10XSTAT92E-GFP (#26198), CycE-lacZ (#30722), (From the Bloomington Drosophila Stock Center, BDSC), *UAS-set1-RNAi* (#40682), *UAS-cic-RNAi* (#103805) (From the Vienna Drosophila RNAi Center, VDRC), *kis*^*LM27*^ and *kis*^*EC1*^ (Melicharek et al., 2008), *NRE-LacZ* (Furriols and Bray, 2001), *Nintra*: *UAS-Notch*^*cdc10*^ is a truncated active version of intracellular Notch, (Brennan et al., 1999), *UAS-LT3-NDam* and *UAS-LT3-NDam-RNAPol II* (Southall et al., 2013) *UAS-LT3-Dam-Pc, UAS-LT3-Dam-HP1a*, *UAS-LT3-Dam-Brm and UAS-LT3-Dam-H1* (Marshall and Brand, 2017), *UAS-cic*^*HA*^ (Jin et al., 2015), pucE69-LacZ (gift from N.Tapon), Upd-LacZ and Upd3.1-LacZ (gift from B. Edgar), UAS-Cbl-L and UAS-Cbl-S (gift from L.M. Pai). The following Gal4 drivers were used: *NRE-GAL4* ; *tubGAL80*^*ts*^ *UAS-GFP* (*NRE*^*ts*^) (Zeng et al., 2010), *esg-GAL4, tubGAL80*^*ts*^ *UAS-GFP* (esg^ts^) (Jiang et al., 2009), *esg-GAL4* UAS-GFP; *Su(H)GBE-GAL80 tubGAL80*^*ts*^ (*esg*^*ts*^*, NREGAL80*) (Wang et al., 2014), *MyoIAGAL4*; *tubGAL80*^*ts*^ *UAS-GFP* (*Myo1A*^*ts*^) (Jiang et al., 2009). *pros*^*voila*^*-Gal4, tub-Gal80ts (pros*^*ts*^*)* (Balakireva et al., 1998).

### Method Details

#### Generation of Transgenic Flies

*kis*_*locus*_ construct containing 524 bp upstream to 12 kb downstream of the *kis* gene was generated starting from *Drosophila* BAC (P[acman] BAC CH321-35E09) that was further altered by recombineering to reduce the genomic size and to add a FLAP cassette N-terminally (amplified from the plasmid R6kam-hNGFP; kindly provided by T. Hyman). The FLAP cassette is composed of green fluorescent protein (GFP), S- and Flag-affinity tags separated by PreScission- and TEV- protease sites. Transgenic flies were generated at Bestgene, Inc. by injection of *attP-9AVK00013*. To generate *UAS-kisL* and *UAS-kisS* transgenic flies, *kis-RA* and *kis-RB* cDNA were respectively amplified from a midgut library and then inserted into the pUASPattB plasmid and tagged with both a 6xHIS N-terminal and a FLAG C-terminal cassette for *kis-RA* and only by a FLAG C-terminal cassette for *kis-RB*. Transgenic flies were generated at Bestgene, Inc. by injection of *attP-3BVK00033* embryos. The mutation of Chd7 K999R, a residue in the highly conserved ATP binding motif of SNF2 superfamily proteins, was shown to prevent its ATPase catalytic activity (Bouazoune and Kingston, 2012). We therefore made the equivalent K2060R mutation in Kismet coding sequence. To generate *UAS-kis-K2060R* transgenic flies, the mutation G>A at 2060th codon was inserted into the pUASP-6His_kis-PA_Flag BAC plasmid by recombineering using the rpsl/neo positive and counterselection system. The final plasmid was tagged with both a 6xHIS N-terminal and a FLAG C-terminal cassettes. Transgenic flies were generated at Bestgene, Inc. by injection of attP-3B-VK00033 embryos. To generate *UAS-Dam-kis* transgenic, *UAST-mCherry-NDam-Myc* sequences amplified from the *pUASTattB-LT3-NDam* plasmid (Southall et al., 2013) were inserted N-terminally to *kis-RA* cDNA *attB* containing vector followed by injection by Bestgene, Inc of *attP2* embryos. To generate *UAS-Dam-trr* transgenic, the *trr* sequence from the ATG to stop codon was obtained starting from *Drosophila* BAC CH322-128O7 that was further altered by recombineering to reduce the genomic size and amplified before insertion C-terminally to *Myc* into *UAST-mCherry-NDam-Myc* plasmid by Gibson Cloning method followed by injection by Bestgene, Inc of P{CaryP}attP2 embryos with the plasmid together with a phiC31 integrase helper plasmid pBS130 as an integrase source.

#### Clonal Analysis and Gal4 Expression

Clones were generated with the Mosaic Analysis with Repressible Cell Marker (MARCM) technique (Lee and Luo, 1999). The following fly stocks were used for MARCM: hsflp122 P[pTub-GAL80] FRT19A; P[pAct-GAL4] (Lin et al., 2008) to produce GFP marked clones on the X chromosome and P[UASGFP]w P[hs-FLP] P[pTub-GAL4] P[UAS-nlsGFP] associated with either FRT40A P[pTub-GAL80] or FRT82B P[pTub-GAL80] to produce GFP marked clones on the second or the third chromosome respectively, w P[hs-FLP]; FRT40A P[pTub-GAL80]; P[UAS-RFP], P[pTub-GAL4] to produce RFP marked clones on the second chromosome and w P[hs-FLP]; FRT40A P[pTub-GAL80]; P[pTub-GAL4] to produce clones expressing UAS-GFP-E2f^1-230^, UAS-mRFP-CycB^1-266^ FUCCI system. MARCM Clones were induced with a heat shock (35 min at 36.5°C) on 3-day-old adult females and were dissected 5, 9, 10, 12 or 30 days after heat shock.

MARCM^ts^ clones were generated using the following stock: w P[hs-FLP] P[pTub-GAL4] P[UAS-nlsGFP]; FRT40A P[pTub-GAL80]; P[pTub-GAL80^ts^]. Crosses were maintained at 18°C, before and after 35 minutes heat shock clone induction at 36.5°C in 3-day-old adult females. 10 days AHS, temperature was shifted to 29°C for 3 days before dissection to allow transgenes expression (UAS-GFP and UAS-N^Act^).

For temporal cell type-specific expression of *kismet RNAi* we used the temperature sensitive inducible UAS-GAL4/GAL80^ts^ system. Crosses and adults were kept at 18°C, the GAL80 permissive temperature. 3-day-old flies were shifted to 29°C for 2, 3 or 10 days to induce RNAi expression.

#### Immunofluorescence and Imaging

As described previously (Bardin et al., 2010), adult female midguts were dissected in PBS and then fixed at room temperature (RT) for 2 hours in 4% paraformaldehyde. Gut were trimmed and incubated in PBS 50% glycerol for 30 minutes before equilibration in PBS 0.1% Triton X-100 (PBT) to clean the lumen. For anti-Notch^ECD^ staining, guts were fixed for 15 min in 4% formaldehyde/heptane followed methanol treatment and rehydration in PBT as described in (Lin et al., 2008). Fixed samples were then washed in PBT for at least 30 min before addition of primary antibodies (overnight at 4°C or 3-5 hours at RT). After at least 30 min wash, secondary antibodies were incubated 3-5 hours before DAPI staining (1 μg/ml) and mounted in 4% N-propyl-galate, 80% glycerol. Polytene immunostainings were performed on L3 larvae salivary glands chromosomes as described in (Zink and Paro, 1995). Salivary glands were fixed in droplet of 45% acetic acid for 3 min. The coverslip was placed onto a poly-L-lysine coated slide and tapped using the tip of a pencil to spread the chromosomes. The quality of the preparations was checked under phase contrast microscope. Slides were next snap-frozen in liquid nitrogen, and coverslip removed. Slides were immediately put in PBS before replacement by blocking solution (1% BSA, 0.5% Triton X100 in PBS) for 1 hour at RT. 50μl of primary antibody in blocking solution was placed onto the chromosome spreads in a humid chamber (1 hour at 4°C). Slides were washed in PBS 0.5% triton for 15 minutes. Secondary antibodies were incubated for 1 hour before DAPI staining (1ìg/ml) and mounted in 4% N-propyl-galate, 80% glycerol.

The following primary antibodies were used: anti-Delta ECD C594.9B (mouse,1:2000, Developmental Studies Hybridoma Bank (DSHB)); anti-GFP (chicken, 1:2000, Abcam), anti-DsRed (rabbit, 1;1000, Clontech), anti-Sanpodo (rabbit, 1:1000; J. Skeath), anti-Notch ECD C458.2H (mouse, 1:100, DSHB), anti-ßGAL (goat, 1:500; Biogenesis), anti-Prospero (mouse, MR1A; 1:1000; DSHB), anti-Pdm1 (rabbit, 1:1000; X. Yang), anti-PH3 (rabbit, 1:1000; Millipore), anti-Kismet DK20 (goat, 1:500; Santa Cruz), anti-Utx, anti-Trr, anti-Lpt and anti-H3K4me1 (Rabbit, 1:500 (Herz et al., 2012)), anti-dpERK (Rabbit, 1:200; Cell Signaling Technology), anti-H3K27me3 (Rabbit, 1:500; Diagenode), anti-H3K27ac (Rabbit, 1:2000; Abcam), anti-EGFR (Mouse, 1:100, Sigma), anti-DH31 (Rabbit, 1:500, J.A Veenstra), anti-LTK2 (Rabbit, 1:1000 J.A Veenstra) and Alexa 647-conjugated phalloidin (1:100, LifeTechnologies). Imaging was performed using Zeiss LSM700 and LSM780 confocal microscopes at the Curie Institute imaging facility with serial optical sections taken at 1 to 1.5-μm intervals (512X512 or 1024X1024) using 20X or 40X oil objectives through the whole-mounted posterior midguts. Representative images are shown in all panels. Super-resolution image was performed with a Structured Imaging Microscope (OMX v3 from Applied Precision-GE Healthcare), equipped with 3 EMCCD, Evolve cameras (Photometrics).

#### DamID-Seq Analysis

In DamID, the fusion of Dam methyl transferase to a DNA associated protein allows the methylation of surrounding GATC sites of DNA, which can be specifically sequenced (Choksi et al., 2006, van Steensel and Henikoff, 2000). In targeted DamID-Seq, cell type-specific low level expression is achieved, thereby allowing in vivo mapping of chromatin associated factors (Southall et al., 2013).

Using the damidseq_pipeline (Marshall and Brand, 2015) reads in fastq files were aligned to the *Drosophila melanogaster* reference genome version 6 using bowtie2 (Langmead and Salzberg, 2012) and alignments were extended to 300 nucleotides or the first GATC site, whichever occurred first.

For all GATC sites in mappable regions read coverage was counted using bedtools coverage (Quinlan and Hall, 2010). GATC sites with fewer than 5 counts on average were discarded. The remaining GATC sites were split into control counts and DamID fusion counts and tested for statistically significant differences using DESeq2 (Love et al., 2014), which also estimates a variance stabilized log_2_ fold enrichment values for each GATC site.

Peaks were called by merging 2 or more consecutive significant GATC sites (adjusted p-value < 0.01, log_2_ fold change > 0). Genes were classified as bound by a protein if 2 consecutive GATC sites within the gene body were occupied with an adjusted p-value < 0.01.

Metaplots were produced using deepTools plotHeatmap (Ramírez et al., 2014) using the DESeq2 output that was converted into bigwig files. Developmental and housekeeping S2 cells enhancers are defined in (Zabidi et al., 2015). Lists of expressed genes and cell type-specific genes were generated from published RNAseq data in the gut (Dutta et al., 2015). All genes with rpkm >1 in the ISC were considered as significantly expressed. Lists of genes enriched in each cell type (ISC, EC, EE) were generated by applying the following criteria: (1) the gene rpkm in one specific cell type is at least 2 times higher than the rpkm in each of the other cell types, (2) the gene rpkm in each other cell type is <2. Lists of EE-enriched genes and EC-enriched genes were merged to generate the list of differentiated cell types-enriched genes.

For the calculation of distribution of Kismet bound GATC sites in the genome in Figure 4A the *Drosophila* gene annotation GTF was downloaded from flybase version 6.13 (Gramates et al., 2017). The GTF file was filtered to retain only 3′UTR coding, 5′UTR coding, exon and gene features. The file was then split into a single file per genomic feature and overlapping features were merged using bedtools. Using bedtools subtract, exonic regions were subtracted from genic regions to obtain intronic regions, and exonic regions were subtracted from overlapping 3′UTR and 5′UTR coding regions. Significantly bound GATC sites were classified as belonging to one of these regions using bedtools intersect.

RNA Pol II occupancy was determined by considering mean ratios (Dam-RNA Pol II/Dam-only) across annotated transcripts using “polii.gene.call” script and false discovery rates (FDR) were assigned (Marshall and Brand, 2015, Southall et al., 2013). Genes with an FDR < 0.01 were used as genes active in ISC (Figures 4J and 6D).

All analysis has been done on Galaxy (Afgan et al., 2016).

A 2D UMAP embedding (McInnes et al., 2018) was created from the log_2_ values estimated by DESeq2. To evaluate the embedding we plotted the log_2_ value for each GATC and each chromatin protein in the UMAP coordinates. We explored effect of varying the n_neighbors, min_dist, n_components and metric parameter and note that varying the parameters results in very similar maps. The parameters used are n_neighbors=30, min_dist=0.0, n_components=2, random_state=42, metric='canberra'.

#### RNAseq Analysis

For transcriptome profiling of sorted ISC, 3-day-old females with either *UAS-GFP* with *UAS-white-RNAi* (control), or with *UAS-kis RNAi* or with *UAS-trr-RNAi* under the control of esg^ts^ NREGal80 were shifted from 18°C to 29°C for 2 days to induce RNAi expression. For each biological replicate (n=3 for *control, n=4 for kis-RNAi, n=3 for trr-RNAi*) midguts from 100 females were dissected in PBS before FACS sorting isolation of ISC GFP+ cells followed by RNA isolation and amplification as described in (Dutta et al., 2013). Reads were quasi-mapped against the *Drosophila* reference transcriptome fasta (Flybase, release 6.13) using Salmon (Patro et al., 2017). Differential gene expression testing was performed using tximportData, RUVseq (Risso et al., 2014) and DESeq2. Genes with an adjusted p-value < 0.01 were considered differentially expressed. All analysis has been done on Galaxy (Afgan et al., 2016).

### Quantification and Statistical Analysis

Image acquisition was followed by data processing with Fiji software and assembled using Adobe Photoshop. Images were processed with a median filter of 1-pixel width before applying Z-stack max projections. All quantification of clonal analysis was limited to the posterior midgut and only clones containing two or more cells (stem cell clones) were scored except for Figure S3J where 1 cell clones were included. Contiguous cells (GFP+ or RFP+) were considered as part of one discrete clone for quantifications. All graphs are scatterplots of raw data to present the full distribution of values observed and all statistical analysis were performed using Prism software. PH3+ cells number per gut was evaluated on the entire midgut (Figure 2D). In Figures 2J and 5H, Kis and Utx staining intensity were quantified within the posterior midgut. The largest nuclear plane for each cell type (esg+ ISC/EB, esg-diploid EE and esg- polyploid Ecs) was determined manually and the average fluorescent intensities of Kismet, Utx and DAPI were calculated with Fiji for these planes. In Figures 5I and 5J, EGFR staining intensity in esg+ cells was quantified per square region within each posterior midgut. The largest plane for each esg+ cell was determined manually in order to measure the cell area and mean fluorescent EGFR intensity with Fiji software and to calculate total EGFR intensity. Statistical analysis were performed using the Graphpad Prism softaware and significance calculated by either two-tailed Mann-Whitney or chi^2^ statistical tests with ns for non-significant, ^∗^ for p<0.05, ^∗∗^ for p<0.01, ^∗∗∗^ for p<0.001 and ^∗∗∗∗^p<0.0001.

### Data and Software Availability

Galaxy workflows, IPython notebooks and UMAP code used in the analysis are available at https://github.com/bardin-lab/kismet-analysis. DamID-Seq and RNA-Seq data have been deposited in the Gene Expression Omnibus (GEO). The accession number for DamID-Seq and RNA-Seq data reported in this paper isGSE128941.

## Supplemental Information

Document S1. Figures S1–S7

Table S1. List of Genes with Kismet Peaks in ISCs from Kis DamID-Seq, Related to Figure 4 This table includes the list of genes with significant Kismet peaks with their Flybase ID, symbol, name, gene start and end and chromosome arm location.

Table S2. RNA Pol II, Polycomb, Brahma, HP1, and Histone H1 Binding Peaks in ISCs from DamID-Seq, Related to Figure 4 This table includes the lists of genes with significant peaks for RNA Pol II (Table S2-1), Polycomb (Table S2-2), Brahma (Table S2-3), HP1 (Table S2-4) and H1 (Table S2-5) with their Flybase ID, symbol, name, gene start and end and chromosome arm location.

Table S3. List of Genes with Significant RNA Pol II Occupancy in ISCs, Related to Figure 4 This table includes the list of genes with significant RNA Pol II occupancy in the ISC (FDR<=0.01) as determined by ISC RNA Pol II-DamID.

Table S4. Trr Binding Peaks in ISCs from Trr DamID-Seq, Related to Figure 6 This table includes the list of genes with significant Trr peaks with their Flybase ID, symbol, name, gene start and end and chromosome arm location.

Table S5. *kis* RNAi and *trr* RNAi RNA-Seq Results, Related to Figure 6 Table S5-1. Corresponds to the list of genes deregulated upon kis knockdown. Table S5-2. Corresponds to the list of genes deregulated upon *trr* knockdown.

Document S2. Article plus Supplemental Information
