# Supplementary material for: Stem Cell Proliferation Is Kept in Check by the Chromatin Regulators Kismet/CHD7/CHD8 and Trr/MLL3/4
Source: Dev Cell. 2019 May 20;49(4):556–573.e6. doi: 10.1016/j.devcel.2019.04.033 (PMC6547167; doi:10.1016/j.devcel.2019.04.033)
Supplement: Document S1. Figures S1–S7 [file mmc1.pdf]

Developmental Cell, Volume *49*

Supplemental Information

Stem Cell Proliferation Is Kept in Check by the Chromatin Regulators Kismet/CHD7/CHD8 and Trr/MLL3/4

Louis Gervais, Marius van den Beek, Manon Josserand, Jérémy Sallé, Marine Stefanutti, Carolina N. Perdigoto, Patricia Skorski, Khallil Mazouni, Owen J. Marshall, Andrea H. Brand, François Schweisguth, and Allison J. Bardin

Supplemental Figures and Legends

Figure S1. Characterization of other *kismet* mutant alleles and rescue experiments, related to Figure 1.

(A, B) Wild-type control (A) and kis^{10D26} mutant (B) MARCM clones, 5 days AHS (GFP, GREEN; Sanpodo (Spdo), RED). **(C-E)** A wild-type clone (C), a kis^{10D26} mutant clone (D), and a kis^{10D26} mutant clone with one copy of genomic rescue construct that partially rescued the *kis* mutant phenotype (E; kis_{locus}), at 9 days AHS. **(F)** Quantification of the cells per clone, and the Delta+ cells per clone from (C-E). **(G-L)** Control clones over-expressing *kisL cDNA* (G), *kisS cDNA* (I), or kis^{K2060R} cDNA a *kis* ATPase dead domain form of *kismet* (K) and kis^{10D26} mutant clones over-expressing *kisL* (H), *kisS* (J) or kis^{K2060R} (L) 9 days AHS (GFP, GREEN; DAPI, BLUE, Delta, RED). **(M, N)** Quantification of cells per clone (M), and Delta+ cells per clone (N) from (G-L). **(O-T)** Mutant clones for kis^{EC1} (O), kis^{LM27} (Q), and kis^{1} (S) alone or over-expressing *kisL cDNA* (P), (R) and (T), respectively, 9 days AHS (GFP, GREEN; DAPI, BLUE; Delta, RED). **(U, V)** Quantification of cells per clone (U), and Delta+ cells per clone (V) from (O-T). Results were compared using a two-tailed Mann-Whitney statistical test. Mean values in RED, error bars= SEM. Scale bars=20 μm.

Figure S2. Kismet is not required in EBs and ECs to control ISC accumulation, related to Figure 2.

(A-E') 2 day *kis* RNAi (BL36597) expression in the ISCs/EBs driven by esg^{ts} (A, A'), in ISCs only driven by esg^{ts}- *NREGAL80* (B, B'), in the ECs driven by Myo^{ts} (C,C'), in the EBs driven by NRE^{ts} (D, D'), or in the EEs driven by $pros^{ts}$ (E, E') was sufficient to deplete Kismet protein (GFP, GREEN; DAPI, BLUE; Kismet, WHITE). Arrows point toward cells with depleted Kismet). **(F, G)** esg^{ts} driven expression of GFP only (F) or with a *kis RNAi* (BL36597) (G) in ISCs and EBs for 10 days at 29°C. Knockdown of *kismet* in ISCs and EBs induced an increase in *esg*+ (GFP, GREEN), and Delta+ cells (RED) compared with control (DAPI, BLUE). **(H, I)** Myo^{ts} driven expression of GFP only (H) or along with a *kis* RNAi (BL36597) (I) in ECs for 10d at 29°C. Knockdown of *kismet* in ECs had no impact on Delta+ cell number (RED) when compared to control (GFP, GREEN;

DAPI, BLUE). **(J, K)** *NRE*^{ts} driven expression of GFP alone (J) or with a *ki*s RNAi (BL36597) (K) in the EB for 10 days at 29°C. *kismet* EB knockdown had no impact on Delta+ cell number (RED) when compared to control (GFP, GREEN; DAPI, BLUE). **(L, M)** *pros*^{ts} driven expression of GFP alone (L) or with a *ki*s RNAi (BL36597) (M) in the EE cells for 10 days at 29°C. *kismet* EE knockdown had no impact on Delta+ cell number (RED) when compared to control (GFP, GREEN; DAPI, BLUE). Scale bars=20 µm.

Figure S3. *kismet* mutant ISCs are able to differentiate in response to Notch signaling activation, related to Figure 3.

(A, B) Notch extra cellular domain (RED) was expressed in most diploid cells in both wild-type control (A), and *kis*^{10D26} mutant (B), clones at 9 days AHS (GFP, GREEN; DAPI, BLUE). **(C-D')** Notch transcriptional reporter activity (NRE-LacZ) was detected in both wild-type control clones (C), and *kis*^{10D26} clones (D), at 9 days AHS (clones outlined in WHITE, GFP, GREEN in C, D; DAPI, BLUE; ßGAL, RED; Delta, GREEN in C', D'). **(E, F)** Quantification of the average number of (NRE+, Delta+) and (NRE-, Delta+) cells per clone (E) and the relative percent of each cell type (F) from (C-D'). **(G)** Experimental set-up: to test the effect of Notch activation on the extra ISCs accumulating in *kis*^{10D26} mutant conditions, wild-type or mutant clones were induced by heat-shock. Flies were maintained for 10 days at 18°C however expression of *UAS-GFP* and *UAS-N*^{Act} (an activated form of Notch) was blocked using GAL80^{ts}, active at 18°C. Flies were either maintained at 18°C or switched to a restrictive temperature of 29°C for 3 days to inactivate GAL80^{ts} thereby allowing GAL4-driven *UAS-GFP* and *UAS-N*^{Act} expression. **(H, I)** Guts 13 days AHS at 18°C containing unmarked wild-type (H), or *kis*^{10D26} mutant clones (I). Note that the *kis*^{10D26} mutant clones could be detected through ISC accumulation marked by Delta (RED; DAPI, BLUE; No GFP and no N^{Act} expression). **(J)** Quantification of total cells per clone, ISCs per clone and ECs per clone after a 3 days switch to 29°C from (J, K). **(K, L)** ISCs were lost upon *UAS-N*^{Act} in wild-type (K), or *kis*^{10D26} mutant clones (L), maintained for 10 days at 18°C AHS before transfer for 3 days at 29°C (GFP, GREEN, DAPI in BLUE and Delta in RED). Most cells in clones had large nuclei,

characteristic of ECs. Results were compared using a two-tailed Mann-Whitney statistical test. Mean values in RED; error bars=SEM. Scale bars=20 μm.

Figure S4. Additional pathways participate to ISC proliferation induced by *kismet* loss, related to Figure 3.

(**A-B')** Wild-type control (A, A'), and kis^{10D26} mutant clones (B, B'), 9 days AHS MARCM clones. As reported in (Biteau et al., 2008) JNK signaling activity, detected by Puc-LacZ (RED in A-B') was detected in wild-type ECs of 15 days old flies but absent in ISCs. While Puc-LacZ was not changed in kis^{10D26} mutant ECs, it was markedly increased in ISCs (clone outlined in WHITE in A', B'; GFP, GREEN; Delta, BLUE; arrowheads point toward ISC). **(C-D')** Wild-type control (C, C'), and kis^{10D26} mutant clones (D, D'), 9 days AHS MARCM clones. JAK/STAT signaling, detected by 10X-STATGFP (GREEN in C-D') was markedly increased in kis^{10D26} mutant clones (clone outlined in WHITE in C', D'; RFP, RED; DAPI, BLUE). **(E)** Quantification of the number of cells per clone, the number of Delta+ cells per clone, and the % of Delta+ cells per clone from 12 day old wild-type control, kis^{10D26} mutant clones, expressing a dominant form of JNK pathway component *basket* (bsk^{DN}), an RNAi construct targeting the JAK/STAT receptor coding gene *dome* (*dome*), expressing a dominant negative form of Insulin receptor (InR^{DN}) or an RNAi construct targeting the Hippo pathway component Yki (*yki RNAi* construct). **(F-M')** Wild-type control (F, F', I, I', L, L'), and kis^{10D26} mutant clones (G G', J, J', M, M'), 3 days AHS MARCM clones. Ligands of JAK/STAT Upd (expressed in ISC and EC) and Upd3 (expressed in the EC) respectively detected using Upd-LacZ (RED in F-G', arrowheads point toward ISC) and Upd3-LacZ (RED in I-J', arrowheads point toward EC) and JNK activity reporter Puc-lacZ (RED in L-M', arrowheads points toward ISC) were not increased in *kismet* mutants at 3 days AHS (clone outlined in WHITE in F', G', I', M'; GFRP, GREEN; DAPI, BLUE). **(H, K, N)** Quantification of the proportion of ISC expressing Upd-LacZ (H), EC expressing Upd3-lacZ (K) and ISC expressing Puc-LacZ (N) in clones from (F-M'). Results compared with two-tailed Mann-Whitney statistical test in (E) and a Chi2 test in (H, K and N). Mean values in RED, error bars= SEM. Scale bars=20 μm.

Figure S5. Kismet distribution on lineage specific genes, related to Figure 4.

(A-A") Super-resolution image of Kismet distribution (Kismet-FLAP tagged kis_{locus} marked by GFP, GREEN) in the nuclei of enterocytes. Kismet was found to be enriched in regions generally lacking the H3K27me3 repressive mark (RED). Scale bar = 5µm. **(B-H)** Wild-type RNAseq, as well as Dam-Kis, Dam-RNA Pol II, Dam-Pc, Dam-Brm, Dam-HP1, Dam-H1 ISC binding profiles and peaks alignments over: (B) the genomic region of the gene *esg,* expressed in the ISCs (having significant RNA Pol II occupancy); (C, D) the genomic region of genes not expressed in the ISCs with low level of Dam-RNA Pol II recruitment (RNA Pol II mean occupancy not significant) though bound by Kismet such as *pros* (C) and *pdm1* (D); (E) the genomic region of genes *ytr* and *eIF6* not bound by Kismet but expressed in ISCs; (F, G) the genomic region of genes not expressed in ISCs *amon* (EE specific), and *pvf3* (EE enriched) (G); (H) the chromosome 3L pericentromeric region at the euchromatin / heterochromatin boundary.

Figure S6. *trr* but not *ash1*, *trx*, *brm* and *set1* chromatin modifiers regulates ISC proliferation similarly to *kismet,* related to Figure 5.

(A-B') Histone H3K27me3 marks (RED) were broadly distributed in cells of wild-type control (A, A') and kis^{10D26} mutant clones (B, B') 9 days AHS; (clones outlined in WHITE; GFP, GREEN; DAPI, BLUE). **(C-G)** Wild-type clones (C), and those expressing an *ash1* RNAi construct (D), trx^{E2} mutant clones (E), or expressing a *brm* RNAi construct (F) or a *set1* RNAi construct (G) at 9 days AHS (clones outlined in WHITE; GFP, GREEN; DAPI, BLUE; Delta, RED). No obvious *kismet*-like ISC accumulation phenotype was detected. **(H, I)** Quantification of cells per clone (H), and the Delta+ cells per clone (I) from (C, D, F). **(J)** Quantification of cells per clone and the Delta+ cells per clone from (K-L'). **(K-L')** Wild-type (K, K'), and trr^{B} mutant clones (L, L'), 9 days AHS MARCM clones; (Clones outlined in WHITE, in K', L'; GFP, GREEN; DAPI, BLUE; Delta, RED). **(M-N')** Histone H3K27ac marks (RED) were broadly distributed in cells of wild-type control (M, M') and kis^{10D26} mutant clones (N, N') 9 days AHS; (clones outlined in WHITE; GFP, GREEN; DAPI, BLUE). **(O-P', R-S')** ISC-specific expression of GFP (O, O'), *Cbl RNAi* (P, P'), *kis RNAi*

(BL34908) (R, R') and *trr RNAi* (S, S') for 3 days at 29°C. Arrows highlight ISC cells. **(Q, T)** Total EGFR fluorescence intensity in GFP+ ISCs (Q), and of the mean EGFR fluorescence intensity per GFP+ ISC (T) in control guts and knock-downs of *Cbl*, *kismet* and *trr* from (O-P', R-S'). Results were compared using a two-tailed Mann-Whitney statistical test. Mean values in red; error bars= SEM. Scale bars=20 µm.

Figure S7. Effects on Histone modifications, Kismet, and Trr COMPASS-like proteins in mutant contexts, related to Figure 7.

(A-B') Wild-type clones (A, A'), and expressing RNAi construct against *trr* (B, B') at 9 days AHS (clones outlined in WHITE; GFP, GREEN; DAPI, BLUE; dpERK, RED) showed an increase in EGFR pathway activity upon knock-down of *trr*. **(C-F)** A control wild-type clone (C), a kis^{10D26} mutant clone (D), a clone expressing *trr RNAi* (E), a kis^{10D26} mutant clone expressing *trr RNAi* (F) at 10 days AHS (GFP, GREEN; DAPI, BLUE; Delta, RED). **(G)** Quantification of clone size and number of Delta+ cells per clone from (C-F). **(H-I')** Kismet protein (RED) in wild-type control clones (H, H'), and *trr RNAi* construct expressing clones (I, I') (clones outlined in WHITE; GFP, GREEN; DAPI, BLUE). **(J-K')** Trr protein (RED) in wild-type control (J, J') and kis^{10D26} mutant clones (K, K'), 9 days AHS (clones are outlined in WHITE; GFP, GREEN; DAPI, BLUE). No changes were found in Kismet or Trr intensity from wild-type to mutant contexts. **(L-O')** Histone H3K4me1 marks (RED) were broadly distributed in wild-type control clones (L, L'), but greatly reduced in both *trr RNAi* (M, M') and *lpt RNAi* construct expressing clones (N, N'). In contrast, kis^{10D26} mutant clones (O, O') showed no obvious reduction in Histone H3K4me1 levels (9 days AHS; clones are outlined in WHITE; GFP, GREEN; DAPI, BLUE). Results were compared using a two-tailed Mann-Whitney statistical test. Mean values in RED; error bars= SEM. Scale bars=20 µm.

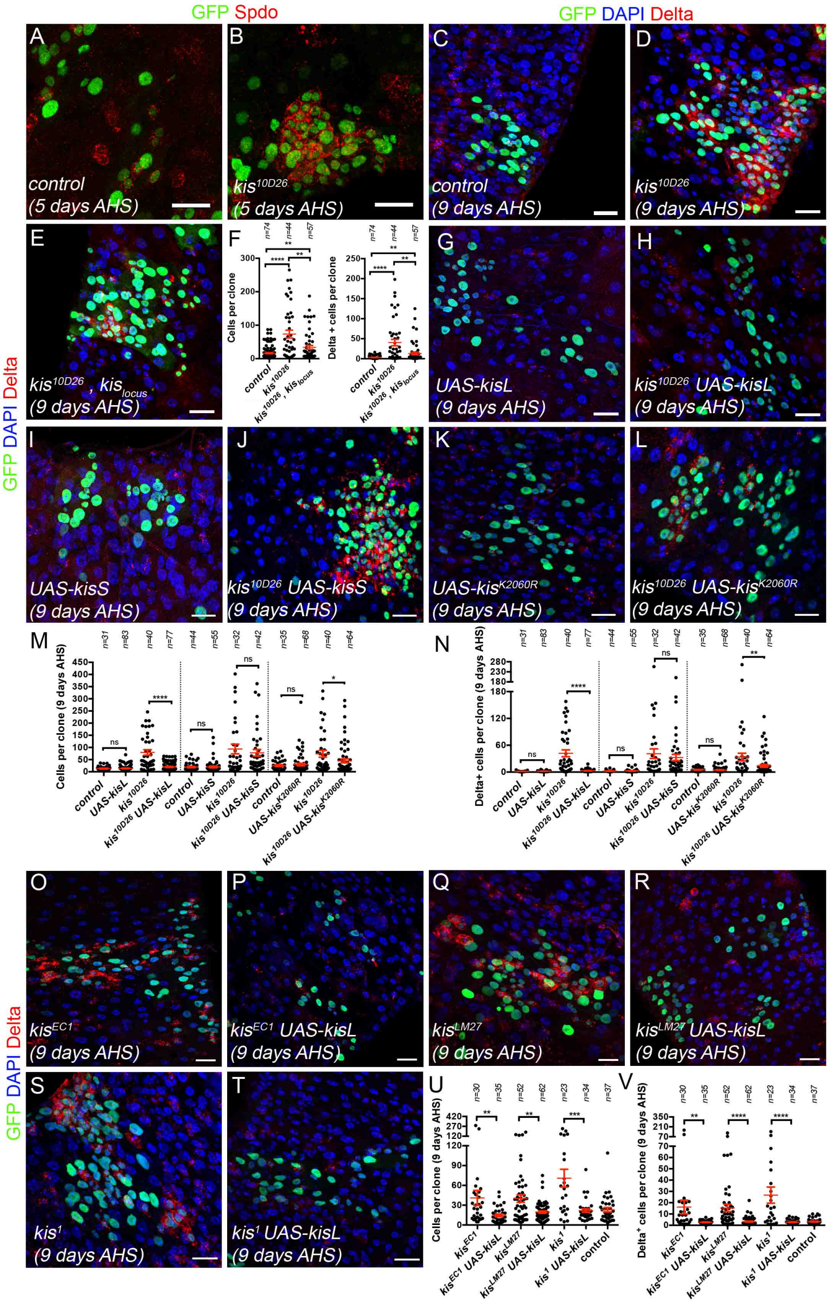

Gervais *et al.*, Fig. S1

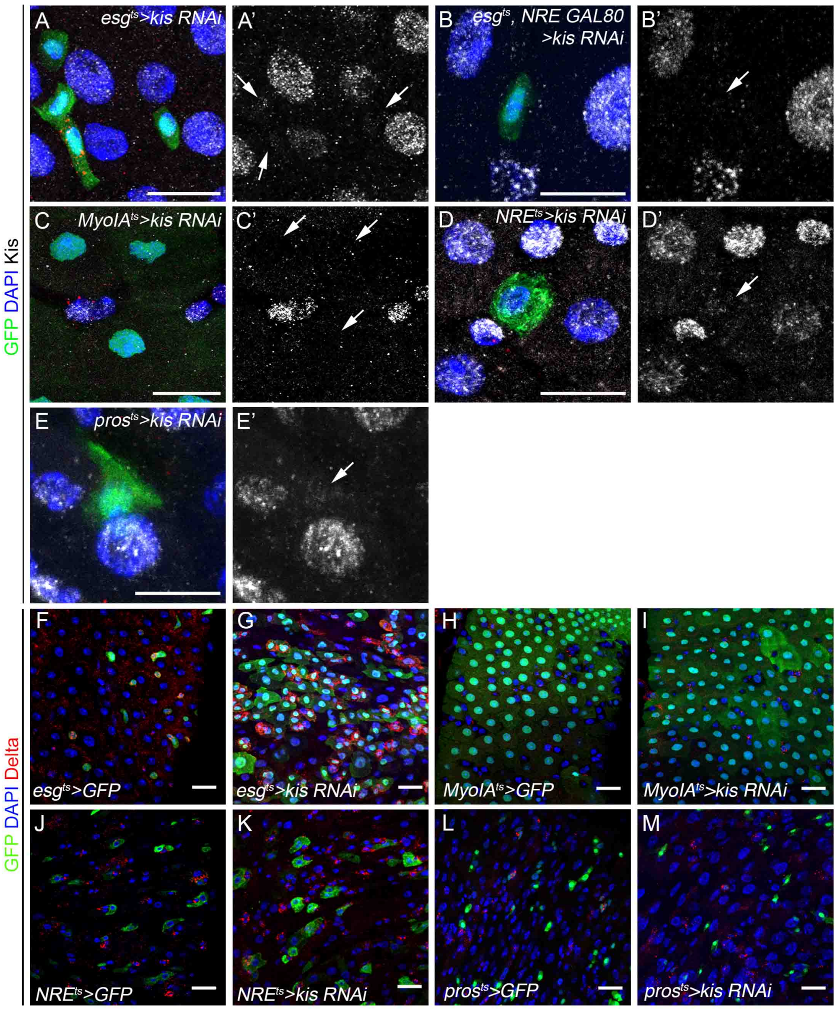

Gervais *et al*. Fig. S2

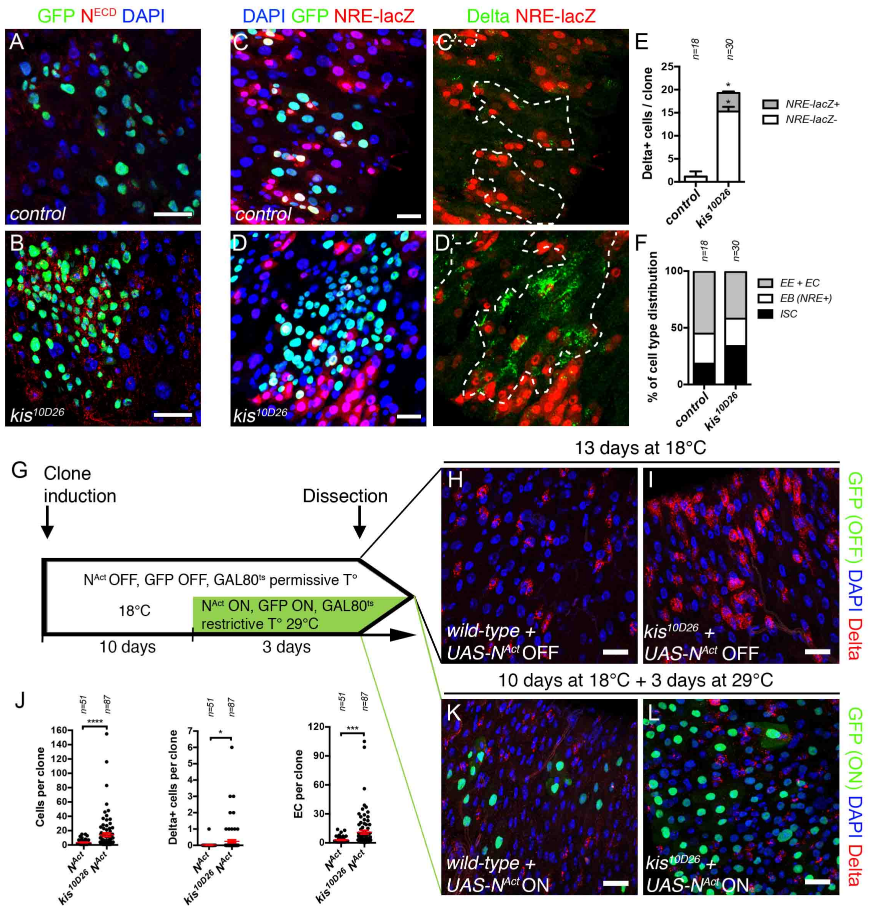

Gervais *et al.*, Fig.S3

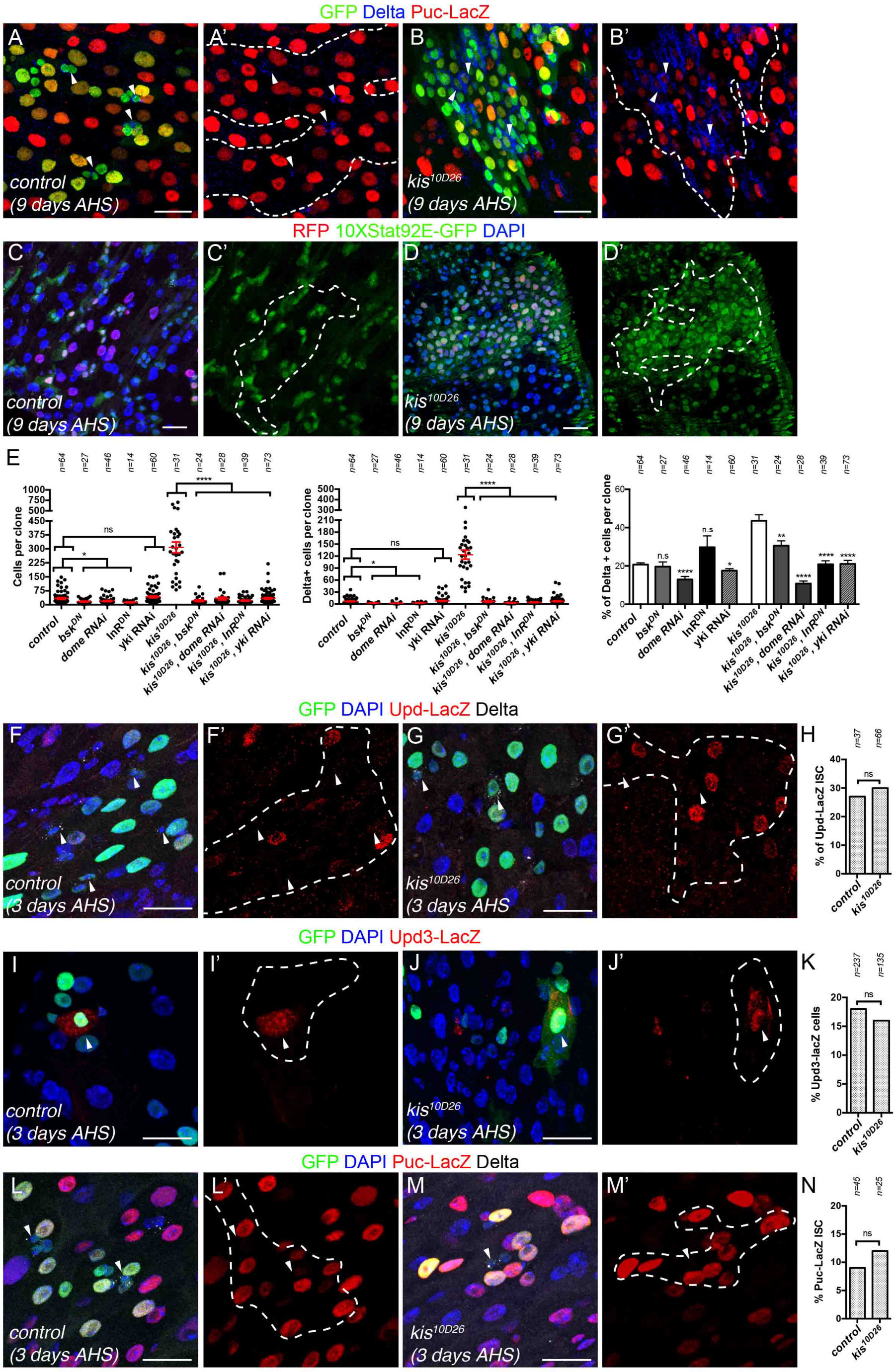

Gervais *et al*. Fig. S4

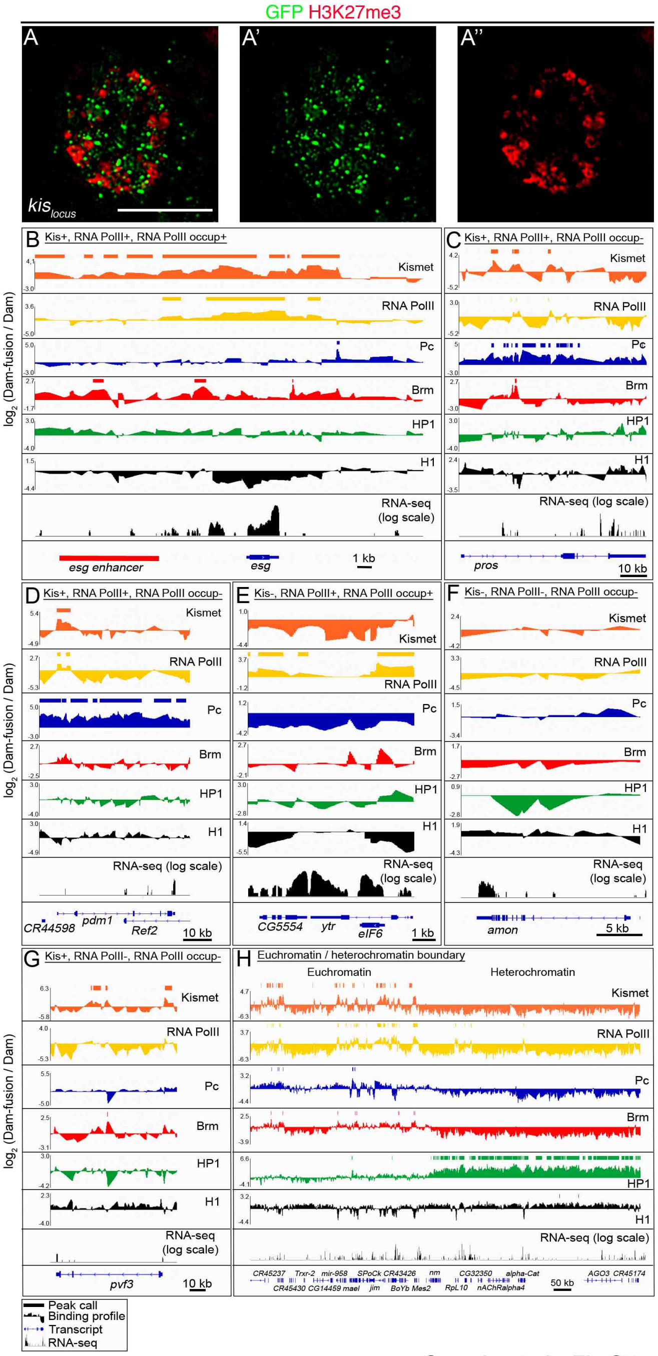

Gervais *et al.*, Fig.S5

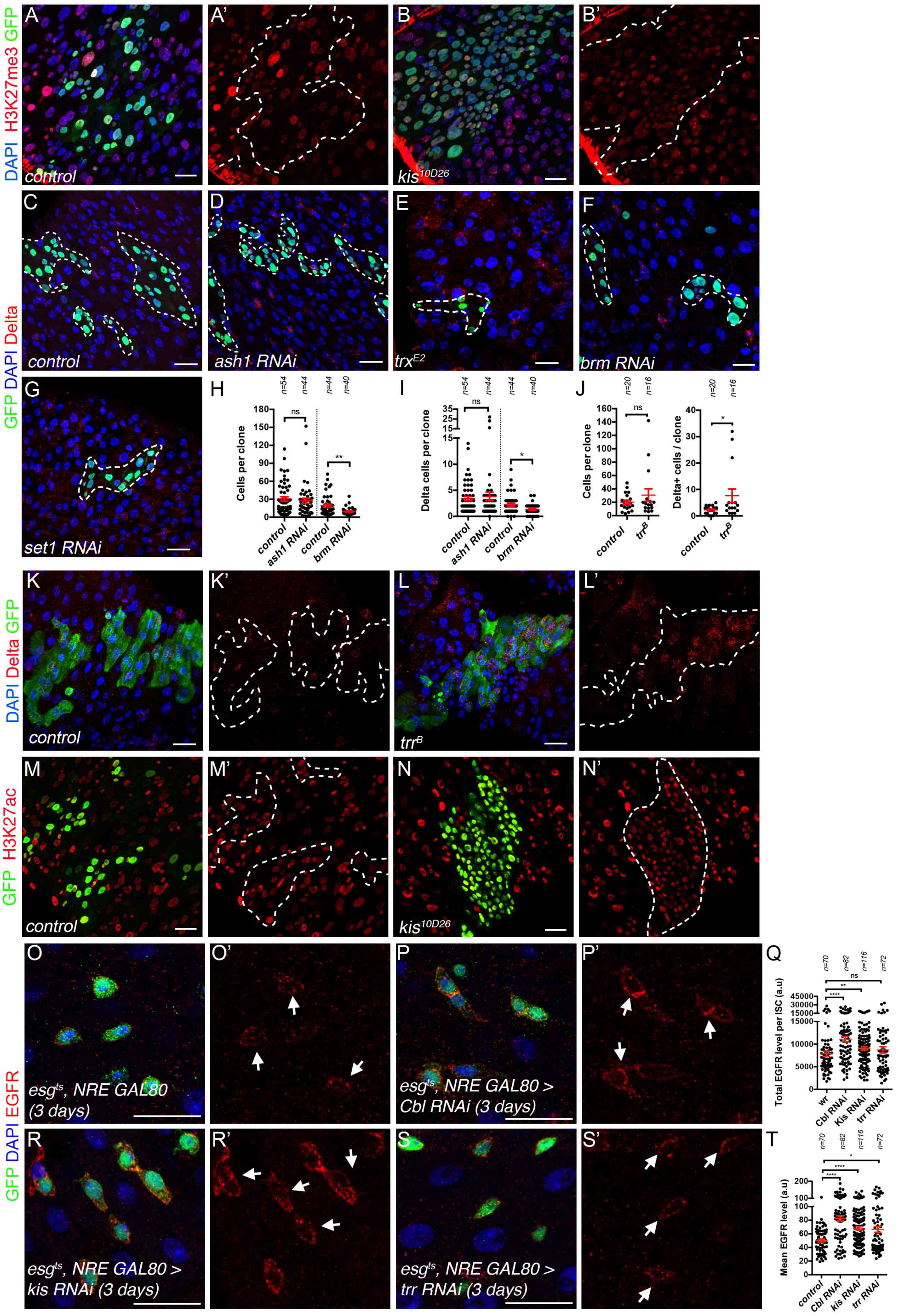

Gervais *et al.*, Fig.S6

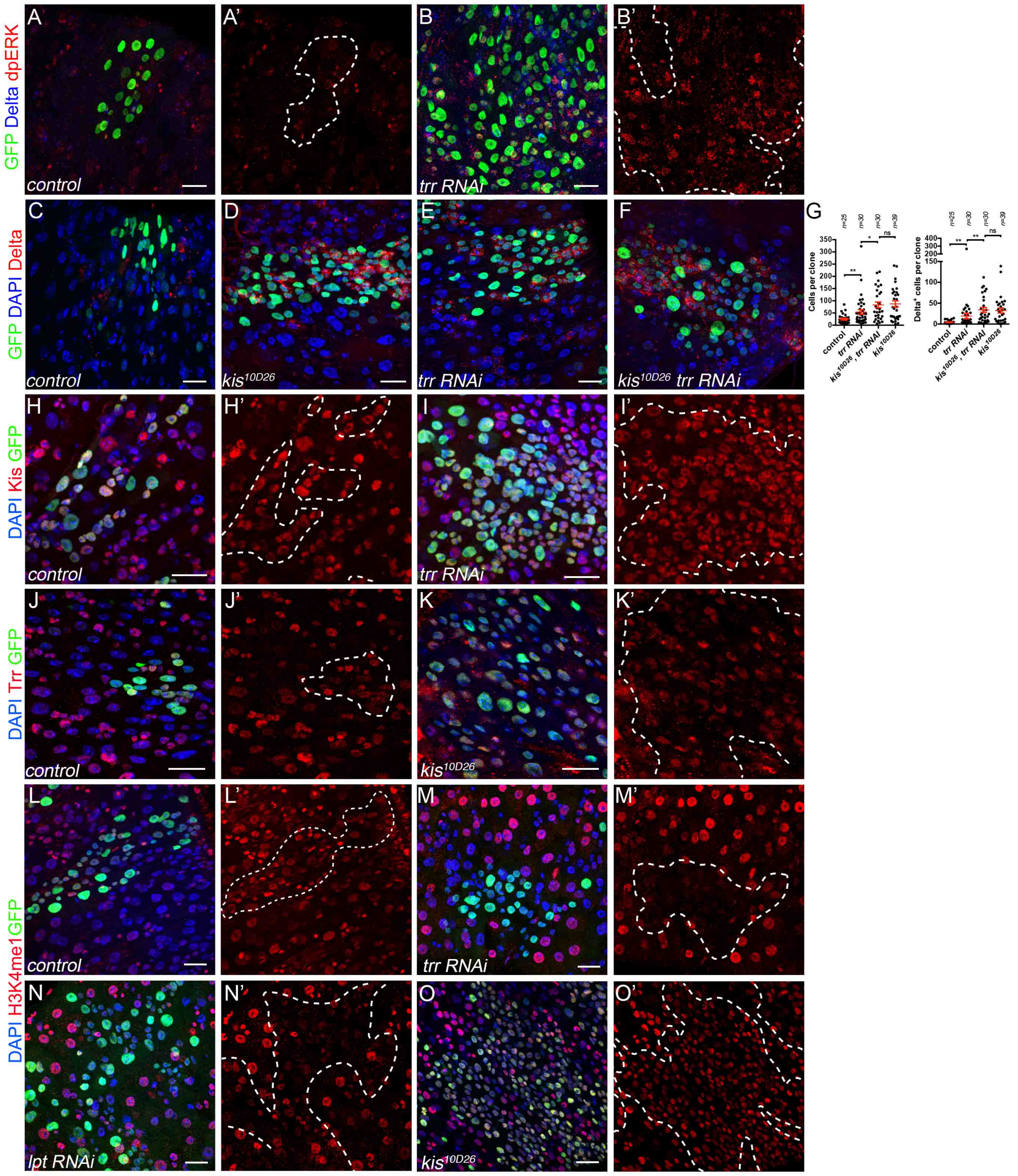

Gervais et al.,Fig. S7
